# Modulation of Auditory Novelty Processing by Dexmedetomidine and Natural Sleep: A Human Intracranial Electrophysiology Study

**DOI:** 10.1111/ejn.70181

**Published:** 2025-07-13

**Authors:** Kirill V. Nourski, Mitchell Steinschneider, Ariane E. Rhone, Rashmi N. Mueller, Matthew I. Banks

**Affiliations:** ^1^ Department of Neurosurgery The University of Iowa Iowa City Iowa USA; ^2^ Iowa Neuroscience Institute The University of Iowa Iowa City Iowa USA; ^3^ Albert Einstein College of Medicine Bronx New York USA; ^4^ Department of Anesthesia The University of Iowa Iowa City Iowa USA; ^5^ Department of Anesthesiology University of Wisconsin Madison Wisconsin USA; ^6^ Department of Neuroscience University of Wisconsin Madison Wisconsin USA

**Keywords:** anesthesia, auditory cortex, averaged evoked potential, consciousness, iEEG

## Abstract

Identifying neural signatures of loss of consciousness is a major goal of neuroscience. The local/global auditory novelty paradigm has been useful in characterizing sensory processing across arousal states. Propofol suppresses responses to long‐term novelty (global deviance, GD) at subhypnotic doses; suppression of responses to short‐term novelty (local deviance, LD) outside auditory cortex may represent a biomarker of loss of consciousness. Dexmedetomidine is an alpha‐2 adrenergic agonist that induces sleep‐like sedation. This study examined whether the changes in auditory novelty processing observed with propofol, a GABA‐ergic agent, also occur with dexmedetomidine and during sleep. Intracranial recordings were obtained in neurosurgical patients undergoing monitoring for refractory epilepsy. Stimuli were vowel sequences incorporating LD and GD. Neural activity was recorded during wakefulness, administration of dexmedetomidine, and sleep and was examined as the averaged evoked potential (AEP) and high gamma (70–150 Hz) power. AEP responses were more broadly distributed than high gamma activity. Results previously observed with propofol were replicated with dexmedetomidine. Subhypnotic doses led to decreased LD effects and a precipitous decline in GD effects. Loss of responsiveness was associated with loss of LD effects outside the auditory cortex. Likewise, daytime sleep was associated with cessation of GD effects and confinement of LD effects to the auditory cortex. Results support the generalizability of changes in auditory novelty processing to dexmedetomidine and sleep. Preservation of LD effects in the auditory cortex indicates that the auditory cortex continues to monitor the environment following loss of responsiveness.

## Graphical abstract

This study examined auditory novelty processing during dexmedetomidine anesthesia and during daytime sleep. Intracranial recordings were obtained in neurosurgical epilepsy patients. In both conditions, loss of consciousness was associated with confinement of local (short‐term) novelty responses to the auditory cortex and was preceded by cessation of global (long‐term) novelty responses. The results align with those previously observed with propofol, supporting shared mechanisms for loss of consciousness.

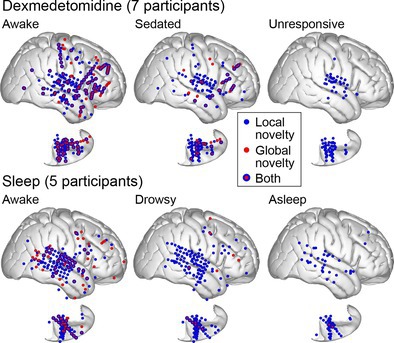

**Abbreviations**
AEP: averaged evoked potential
ACC: anterior cingulate cortex
AG: angular gyrus
Amyg: amygdala
BIS: bispectral index
Caud: caudate nucleus
CingMA: middle‐anterior portion of cingulate gyrus
CingMP: middle posterior portion of cingulate gyrus
CingPD: postero‐dorsal portion of cingulate gyrus
CingPV: postero‐ventral portion of cingulate gyrus
EEG: electroencephalography
ERBP: event‐related band power
FG: fusiform gyrus
FOp: frontal operculum
FP: frontal pole
GD: global deviance
GR: gyrus rectus
GS: global standard
HG: Heschl's gyrus
HGAL: anterolateral portion of Heschl's gyrus
HGPM: posteromedial portion of Heschl's gyrus
Hipp: hippocampus
iEEG: intracranial EEG
IFG: inferior frontal gyrus
IFGop: IFG pars opercularis
IFGor: IFG pars orbitalis
IFGtr: IFG pars triangularis
IPS: intraparietal sulcus
ITG: inferior temporal gyrus
InsA: anterior portion of insula
InsP: posterior portion of insula
LD: local deviance
LGD: local/global deviant
LingG: lingual gyrus
LME: linear mixed effects
LOC: loss of consciousness
LOR: loss of responsiveness
LS: local standard
MEG: magnetoencephalography
MFG: middle frontal gyrus
MMN: mismatch negativity
MNI: Montreal Neurological Institute
MOG: middle occipital gyrus
MTGA: anterior portion of MTG
MTGM: middle portion of MTG
MTGP: posterior portion of MTG
OAA/S: Observer's Assessment of Alertness/Sedation
OG: orbital gyri
OR: odds ratio
ParaCL: paracentral lobule
PHG: parahippocampal gyrus
PMC: premotor cortex
POp: parietal operculum
PostCG: postcentral gyrus
PP: planum polare
PreCG: precentral gyrus
PreCun: precuneus
PT: planum temporale
ROI: region of interest
SFG: superior frontal gyrus
SMG: supramarginal gyrus
STG: superior temporal gyrus
STGA: anterior portion of STG
STGM: middle portion of STG
STGP: posterior portion of STG
STS: superior temporal sulcus
SubCG: subcallosal gyrus
SubInn: substantia innominata
TP: temporal pole

## 1 Introduction

Identifying neural signatures that can distinguish conscious from unconscious patients with high sensitivity and specificity is a major goal of clinical neuroscience. Clinically relevant conditions include sedation and loss of consciousness (LOC) associated with general anesthesia and disorders of consciousness including delirium and coma. Recent commentaries on the classification of disorders of consciousness stress the need for objective measures beyond those that solely rely on behavioral assessments (Bayne et al. 2017; Bernat 2017). Similar needs for objective measures of neural activity exist for the prevention of intraoperative awareness, a serious albeit extremely rare complication of general anesthesia (Avidan et al. 2011).

Loss of responsiveness (LOR) (and presumably LOC) associated with general anesthesia or sleep is characterized by diminished cortical sensory processing and disruption of auditory predictive coding (Portas et al. 2000; Liu et al. 2012; Wilf et al. 2016). This disruption has been hypothesized to represent a signature of LOC (e.g., Bekinschtein et al. 2009; Mashour 2014; Uhrig et al. 2016; Nourski et al. 2018b; Sanders et al. 2021). The local/global deviant (LGD) paradigm engages auditory predictive coding on multiple time scales and has been used to characterize sensory processing across states of arousal (e.g., Bekinschtein et al. 2009; Strauss et al. 2015).

In the LGD paradigm, a change within a local acoustic environment (hundreds of milliseconds) constitutes short‐term novelty or local deviance (LD). A change in the pattern of sound sequences on the order of seconds in the same paradigm represents long‐term novelty or global deviance (GD) (Bekinschtein et al. 2009). Physiological responses elicited by LD and GD include the mismatch negativity (MMN) and the P3b novelty response, respectively (Näätänen and Alho 1995; Kok 2001). These responses are measured with non‐invasive methods [electroencephalography (EEG) or magnetoencephalography (MEG)] which lack sufficient spatial resolution to determine the precise cortical origins of novelty processing and their spatiotemporal dynamics. This limitation hampers the identification of neural signatures of LOC that may be clinically useful. Additionally, anesthetic agents may produce non‐uniform changes in blood flow and metabolic activity throughout the brain (Heinke and Schwarzbauer 2002). This in turn may bias the results of non‐invasive neuroimaging studies examining the effects of anesthesia on neural activity.

Intracranial recordings offer the advantage of high temporal and spatial resolution for a better characterization of mechanisms associated with predictive coding. Our previous intracranial electroencephalography (iEEG) studies have characterized modulation of auditory novelty processing associated with sedation and LOR induced by propofol (Nourski et al. 2018b, 2021b). Cortical responses to GD were highly sensitive to subhypnotic doses of propofol, whereas LD responses were more resilient and persisted within the auditory cortex following LOR. Importantly, the transition from sedation to LOR was paralleled by the loss of responses to LD outside the canonical auditory cortex, suggesting that this loss may represent a biomarker of LOC.

Propofol exerts its effects on auditory novelty detection via actions at GABA_A_ receptors, or via other molecular targets in the brain. Results in aged rodents suggest that deficits in GABA_A_ receptor‐mediated inhibition contribute to impaired novelty detection (de Villers‐Sidani et al. 2010; Caspary et al. 2013). Indeed, GABA_A_ receptors regulate neuronal excitability, adaptation, and NMDA receptor‐mediated plasticity (Pérez‐González et al. 2012; Askew and Metherate 2016; Schulz et al. 2018), all critical components of novelty detection (Malmierca et al. 2014). Additionally, propofol and other general anesthetics can modulate cortico‐cortical connectivity independently of these agents' effects on GABA_A_ receptors (Hao et al. 2020).

The motivation for the present study was twofold. First, it remains an open question whether the effects of the anesthetic agent, propofol, on auditory predictive processing are drug‐specific based on its action upon GABA_A_ receptors or are more broadly representative of LOC. The present iEEG study thus examined whether the changes in auditory predictive processing observed with propofol could be generalized to another drug used for sedation, dexmedetomidine. Dexmedetomidine is a potent alpha‐2 adrenergic agonist (Nguyen et al. 2017) that may additionally modulate GABAergic transmission (Tang et al. 2025). At equivalent sedative doses, the effects of the two drugs on neural activity are not identical, as exemplified by differential modulation of visual and motor cortical oscillations (Saxena et al. 2021). The importance of investigating dexmedetomidine is highlighted by the fact that this agent is a safer sedative than propofol in certain clinical scenarios such as prolonged administration in intensive care units or use in very young patients. This leads to the increased use of dexmedetomidine in pediatric and adult patients (Lee 2019; Chen et al. 2024, Singh and Anjankar 2022). The second reason we undertook this study is due to the findings that dexmedetomidine induces sedation that is neurophysiologically similar to non‐rapid eye movement sleep and during which the patient can remain arousable and has hemodynamic and respiratory stability (Akeju et al. 2018; Nelson et al. 2003). Hence, the present study tested the hypothesis that the similarity of dexmedetomidine‐induced sedation to natural sleep would be paralleled by concordant changes in auditory predictive processing.

## 2 Methods

### 2.1 Ethics Statement

Research protocols were approved by the National Institutes of Health and the University of Iowa Institutional Review Board (protocols #200112047 “Human Brain Physiology Research,” #201911084 “Research of Physiology of Human Brain,” and #201804807 “Mechanisms of Consciousness Research”). Written informed consent was obtained from all participants. Research participation did not interfere with acquisition of clinically necessary data, and participants could rescind consent for research at any time without interrupting their clinical management.

### 2.2 Participants

The study included 11 adult neurosurgical patients (5 female; age 18–46 years old, median age 36.5 years old) diagnosed with drug‐resistant epilepsy. The patients were implanted with intracranial electrodes to identify resectable seizure foci. Participants' age, sex, electrode coverage, and seizure focus data are summarized in Table S1. Seven of the participants contributed to the dexmedetomidine data set; one of those patients plus the remaining four constituted the sleep cohort (see *Stimuli and Procedure* below). All participants were right‐handed except R456 and R413, who were left‐handed. All had left language dominance as determined by Wada tests except R413, who was right hemisphere‐dominant. The letter prefix of the participant code denotes the hemisphere of electrode implantation over the presumed side of seizure foci: L = left (*N* = 5); R = right (*N* = 5); B = bilateral (*N* = 1). Most participants had, to varying degrees, bilateral coverage of the brain. All participants underwent preoperative audiometric and neuropsychological evaluations, and no hearing or cognitive deficits that should impact the findings presented in this study were identified. The participants were tapered off their antiseizure medications following electrode implantation and had their medication regimens reinstated by the end of the monitoring period (19 days in L585, 21 days in R728, 14 days in all other participants), prior to the electrode removal surgery.

### 2.3 Stimuli and Procedure

Auditory stimuli were quintuples of vowels/ɑ/ and /i/, presented in an LGD paradigm (Bekinschtein et al. 2009; Nourski et al. 2018a; Figure S1). The vowels were excised from steady‐state vocalic portions (100 ms) of consonant‐vowel stimuli /hɑd/ and /hid/, spoken by a female talker (fundamental frequency 232 Hz and 233 Hz, respectively) (Hillenbrand et al. 1995). The vowels were normalized to the same root‐mean‐square amplitude and gated with 5 ms on/off ramps (Figure S1a). On each trial, four identical vowels, separated by 50‐ms intervals, were presented, followed by either the same or different fifth vowel (Figure S1b). This within‐quintuple difference constituted short‐term (local) deviance: stimuli /ɑɑɑɑɑ/ and /iiiii/ were local standards (LS), while /ɑɑɑɑi/ and /iiiiɑ/ were local deviants (LD). Blocks were 11 min long and contained four sequences, with the order of the sequences randomized across blocks (Figure S1c). Global standards (GS) were established by presenting 10 identical habituation trials at the beginning of each of the four sequences. Thus, in any block, the GS could be an LS or an LD, and similarly, the GD could be either LD or LS.

In participants R456, B457, R458, and R413, the first 10 trials in each sequence established the GS condition (e.g., /ɑɑɑɑɑ/for Sequence 1; habituation trials), followed by 80 GS and 20 GD test trials, presented in a pseudorandom order. The difference in presentation frequency of GS and GD stimuli constituted the long term (global) deviance, and the identity of the GD stimulus changed across the four sequences within each block. The GD target detection task was explained to the participants by a member of the research team beforehand as follows: “Press the button every time you hear the sound sequence change.” A modification of this paradigm was used in participants L525, L625, R720, R728 (dexmedetomidine experiment), and L372, L514, L585 (sleep experiment), wherein each of the four sequences was preceded by a 15‐s instruction (“Press the button every time you hear this sound … Once again, press the button every time you hear this sound …”). In these participants, the number of GS and GD test trials in each sequence was reduced to 72 and 18, respectively, to maintain the same 11‐min duration of the recording block.

Stimuli were presented by a TDT RZ2 processor (Tucker‐Davis Technologies, Alachua, Florida) and delivered at a comfortable level (60–65 dB SPL) diotically via insert earphones (ER4B, Etymotic Research) enclosed in custom‐fit earmolds. The intertrial interval varied within a Gaussian distribution (onset‐to‐onset mean 1500 ms, standard deviation 10 ms) to reduce heterodyning in the recordings secondary to the 60 Hz power line noise. The participants were instructed to operate the response button with the hand ipsilateral to the hemisphere with predominant electrode coverage. This was done to minimize contributions of activity reflecting motor planning and execution, as well as somatosensory responses associated with the button press, to recorded neural responses to the auditory stimuli.

Dexmedetomidine experiments were conducted in the operating room immediately prior to and during induction of general anesthesia for electrode removal. The time course of the dexmedetomidine experiment in each participant is shown in Figure S2a. Each experiment included three 11‐min blocks, separated by 9‐min intervals used to collect resting state iEEG data as part of a companion study (Krause et al. 2025). Following the completion of the first (pre‐drug) block, a bolus dose of dexmedetomidine (0.25 μg/kg) was administered, followed by infusion at a rate between 0.2–0.8 μg/kg/h (Alaris Pump, BD, Maplewood, MO). Following the completion of the second block, the dexmedetomidine infusion rate was increased to 0.8–4 μg/kg/h. Dexmedetomidine was the sole sedative drug administered to the participants during the experiment. The infusions were administered by an attending anesthesiologist using standard respiratory and cardiac monitoring. None of the infusions had to be interrupted or terminated for the patients' safety.

The depth of sedation was evaluated before and after each block using the Observer's Assessment of Alertness/Sedation (OAA/S) scale (Chernik et al. 1990). This assessment included tests of responsiveness (calling the subject's name), speech repetition (asking the subject to repeat the sentence, “The quick brown fox jumps over the lazy dog”), facial expression (the degree of facial relaxation), and eyes (the ability to focus and ptosis), each scored on a scale from 1 to 5. The composite OAA/S score, ranging from 5 (“alert”) to 1 (“deep anesthesia”), was defined as the lowest level indicated by any of the four assessment categories. The depth of sedation was additionally assessed using bispectral index (BIS; BIS Complete 4‐Channel Monitor; Medtronic, Fridley, Minnesota) (Gan et al. 1997). BIS, an index on a scale of 0 to 100 derived from the electroencephalogram, was recorded continuously throughout each experiment and was manually logged on a minute‐by‐minute basis (lower BIS values indicate more sedation).

For the purposes of analyses, three states were defined in each participant: awake (before administration of dexmedetomidine), sedated (during administration of dexmedetomidine, average OAA/S score > 2), and unresponsive (during administration of dexmedetomidine, average OAA/S score ≤ 2) (Figure S2b). LOR to command, as determined by OAA/S, was assumed to indicate LOC (Nourski et al. 2018b; Banks et al. 2020), though we cannot exclude the possibility that participants were dreaming and thus were in a state of disconnected consciousness (Scheinin et al. 2021; Casey et al. 2022).

Sleep experiments were conducted during the daytime in a dedicated, electrically shielded suite in The University of Iowa Clinical Research Unit. The room was quiet, with lights dimmed, and participants were comfortably reclining in a hospital bed. They were instructed to perform the GD target detection task during the first block, continue to perform it during the subsequent blocks if they felt awake, but “let go” if they felt drowsy. For the purposes of analyses, three states were defined in each participant: awake (the first block, while all participants performed the task), drowsy (the second block), and asleep (the last block, when the participants did not press the response button, had their eyes closed, and did not respond to the research team member entering the room after the block). Sleep was confirmed by the presence of sleep spindles, seen as a peak in the alpha band (~12 Hz) in frontal recording sites (Andrillon et al. 2011). The time course of the sleep experiment in each participant is shown in Figure S2c.

### 2.4 Electrophysiological Recordings

Recordings were obtained using subdural or depth electrodes implanted based on clinical requirements, as determined by the team of epileptologists and neurosurgeons at the University of Iowa Hospitals and Clinics. Details of electrode implantation, recording, and iEEG data analysis have been described previously (e.g., Nourski and Howard 3rd 2015). Electrode arrays were manufactured by Ad‐Tech Medical (Racine, Wisconsin) or PMT (Chanhassen, Minnesota). Subdural arrays, implanted in 5 participants out of 11, consisted of platinum‐iridium discs (2.3 mm diameter, 5–10 mm inter‐electrode center‐to‐center distance), embedded in a silicon membrane. Stereotactically implanted depth arrays included between 4 and 14 cylindrical contacts along the electrode shaft, with 2.2–10 mm inter‐electrode spacing. A subgaleal electrode, placed over the cranial vertex near midline, was used as a reference in all participants.

Data acquisition was done by a TDT RZ2 real‐time processor (Tucker‐Davis Technologies, Alachua, Florida) or a Neuralynx Atlas System (Neuralynx, Bozeman, Montana). Recorded data were amplified, filtered (0.7–800 Hz bandpass, 5 dB/octave rolloff for TDT‐recorded data; 0.1–500 Hz bandpass, 12 dB/octave rolloff for Neuralynx‐recorded data), and digitized at a sampling rate of 2034.5 Hz (TDT) or 2000 Hz (Neuralynx).

### 2.5 Analysis

To examine the possible effect of OAA/S assessments on arousal, BIS values recorded immediately before and after each block (see Figure S1a) were compared using a linear mixed effects model:

$$\mathrm{BIS}\sim {\text{PrePost}}^{*}\text{Assessment}\#+\left(1|\text{Participant}\right),$$

where

*PrePost* is the fixed effect of the binary variable representing whether BIS measurement was made before or after OAA/S assessment;

*Assessment#* is the fixed effect of the order of the OAA/S assessment (1 = pre‐sedated block; 2 = post‐sedated; 3 = pre‐unresponsive; 4 = post‐unresponsive);

*Participant* is the random effect of the participant.

Anatomical localization of recording sites was based on post‐implantation structural magnetic resonance imaging (MRI) and computed tomography (CT) data. Images were first aligned with pre‐operative T1 MRI scans using linear co‐registration implemented in FSL (FLIRT) (Jenkinson et al. 2002). Accuracy of electrode localization within the pre‐operative MRI space was refined using three‐dimensional non‐linear thin‐plate spline warping to correct for post‐operative brain shift and distortion (Rohr et al. 2001). The warping was constrained within 50–100 control points manually selected throughout the brain, which were visually aligned to anatomical landmarks in the pre‐ and post‐implantation scans.

Each recording site was assigned to one of four groups based on anatomical reconstructions of electrode locations in each participant, a simplified version of a scheme used previously in Banks et al. (2023) and Nourski et al. (2024):

1 Auditory:

- Heschl's gyrus (HG), including its posteromedial and anterolateral portions (HGPM, HGAL)
- Planum polare (PP)
- Planum temporale (PT)
- Superior temporal gyrus (STG), including its middle and posterior portions (STGM, STGP)

2 Auditory‐related:

- Middle temporal gyrus (MTG), including its anterior, middle, and posterior portions (MTGA, MTGM, MTGP)
- STG, anterior portion (STGA)
- Superior temporal sulcus (STS)
- Angular gyrus (AG)
- Supramarginal gyrus (SMG)

3 Prefrontal:

- Anterior cingulate cortex (ACC)
- Frontal pole (FP)
- Inferior frontal gyrus (IFG), including pars opercularis, orbitalis, and triangularis (IFGop, IFGor, IFGtr).
- Middle frontal gyrus (MFG)
- Orbital gyri (OG)
- Superior frontal gyrus (SFG)

4 Other areas:

- Amygdala (Amyg)
- Hippocampus (Hipp)
- Parahippocampal gyrus (PHG)
- Paracental lobule (ParaCL)
- Postcentral gyrus (PostCG)
- Precental gyrus (PreCG)
- Caudate nucleus (Caud)
- Cingulate gyrus, including its middle anterior, middle posterior, posterodorsal, and posteroventral portions (CingMA, CingMP, CingPD, CingPV)
- Fusiform gyrus (FG)
- Gyrus rectus (GR)
- Intraparietal sulcus (IPS)
- Inferior temporal gyrus (ITG)
- Insula, including its anterior and posterior portions (InsA, InsP)
- Lingual gyrus (LingG)
- Middle occipital gyrus (MOG)
- Premotor cortex (PMC)
- Precuneus (PreCun)
- Subcallosal gyrus (SubCG)
- Substantia innominate (SubInn)
- Temporal pole (TP)
- Opercular cortex, including frontal and parietal operculum (FOp, POp)

**FIGURE 1:**
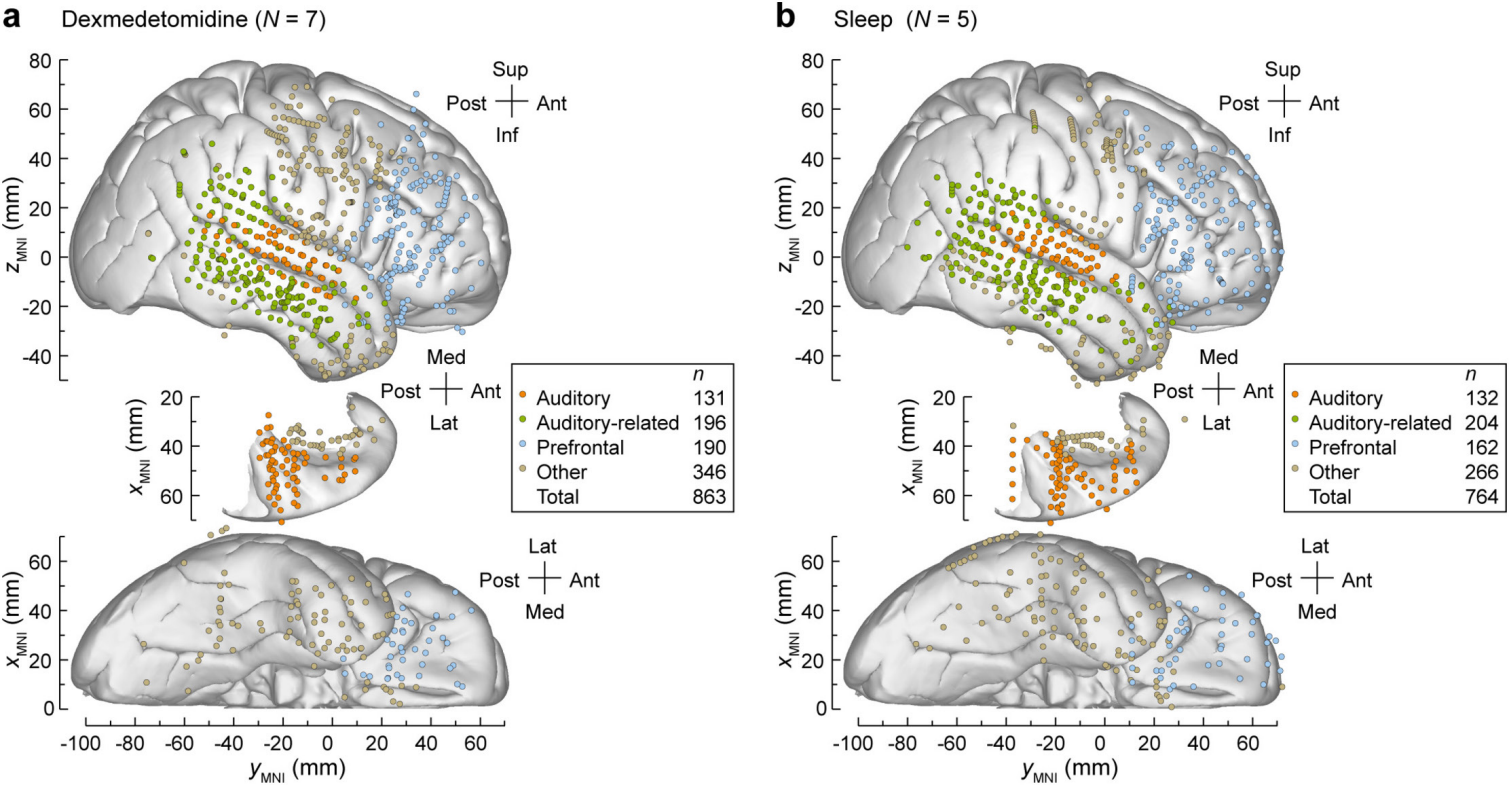
Summary of electrode coverage for participants in dexmedetomidine (7 participants (a)) and sleep (5 participants (b)) experiments. Locations of recording sites, determined for each participant individually and color‐coded by region‐of‐interest (ROI) group, are plotted in MNI coordinate space and projected onto the Freesurfer average template brain for spatial reference. Projections are shown in the lateral, top‐down (superior temporal plane), and ventral views (top to bottom). Numbers of sites in each ROI group across all participants for each experiment are denoted in the legends. Sites in the amygdala, caudate nucleus, cingulate cortex, frontal operculum, hippocampus, lingual gyrus, parietal operculum, paracentral lobule, precuneus, subcallosal gyrus and substantia innominata were analyzed but not shown.

Electrode coverage in the dexmedetomidine and sleep data set is summarized in Figure 1a,b, respectively. Assignment of recording sites to regions of interest (ROIs) and their subsequent grouping was based on automated parcellation of cortical gyri, implemented in the FreeSurfer software package (Destrieux et al. 2010, 2017) and then confirmed by visual inspection of anatomical reconstruction data. For recording sites in HG, delineation of the border between core auditory cortex and adjacent non‐core areas (HGPM and HGAL, respectively) was performed in each participant using physiological criteria (Brugge et al. 2009; Nourski et al. 2016). STG was subdivided into posterior and middle non‐core auditory cortex ROIs (STGP and STGM), and an auditory‐related anterior ROI (STGA) using the transverse temporal sulcus and ascending ramus of the Sylvian fissure as boundaries. The insula was subdivided into InsP and InsA (long and short insular gyri, respectively) (Zhang et al. 2019). MTG was divided into MTGA, MTGM, and MTGP by dividing the gyrus into three approximately equal‐length thirds. ACC was identified by automatic parcellation in FreeSurfer and was considered part of the prefrontal ROI group (i.e., separately from the rest of the cingulate gyrus). Recording sites identified as seizure foci or characterized by excessive noise, and depth electrode contacts localized outside cortical gray matter were excluded from analyses.

Electrophysiological data analysis was performed using custom software written in MATLAB (MathWorks, Natick, Massachusetts). Recordings were downsampled to 1000 Hz for computational efficiency and de‐noised using a demodulated band transform‐based procedure (Kovach and Gander 2016). Voltage deflections exceeding five standard deviations from the within‐block mean for each recording site were considered artifacts, and trials containing such deflections were excluded from further analysis.

Analysis of iEEG data focused on local field potentials, examined in the time domain as averaged evoked potentials (AEPs), and high gamma (70–150 Hz) event‐related band power (ERBP). Single‐trial local field potential waveforms were baseline‐corrected by subtracting mean voltage in the 100 ms time window immediately preceding stimulus onset and averaged to obtain the AEP. High gamma ERBP was calculated by bandpass filtering the iEEG signal (300th order finite impulse response filter, 70–150 Hz passband), followed by Hilbert envelope extraction, log transformation, and, for each of the four experimental sequences, normalization to the mean power over the entire duration of the sequence. AEPs and high gamma ERBP waveforms were smoothed using a lowpass Butterworth filter (4th order, 30 Hz cutoff).

AEPs and ERBP representing responses to the first four vowels prior to the onset of deviance defined by the fifth vowel, were computed by averaging single‐trial waveforms across all trials. Responses to local and global auditory novelty were computed as the difference between averaged responses to standard and deviant trials. Throughout the manuscript, the following nomenclature is used to refer to these responses. “LGD effects” refers to both LD and GD effects, measured either as the AEP or high gamma ERBP. “LD effects” and “GD effects” refer to local and global novelty responses, respectively, measured as the AEP or high gamma ERBP. “The AEP LD effect,” “the AEP GD effect,” “the high gamma LD effect,” and “the high gamma GD effect” refer to the specific type of novelty effect as measured by the specific neural response.

Significance of LD and GD effects was established within 0–800 ms following the onset of the fifth vowel to exclude responses to the next trial which began at ~900 ms. Non‐parametric cluster‐based permutation tests (Maris and Oostenveld 2007; Nourski et al. 2018a, 2021a) were based on grouping adjacent time samples that exhibited a significant difference between standard and deviant trials. The cluster statistics were constructed by first computing two‐sample *t*‐statistics across all time points for each recording site. For each time point, *t*‐values were compared to a threshold corresponding to the 1st percentile tail of the *T*‐distribution. The threshold was the 99.5th percentile for two‐tailed tests for AEP and 99th percentile for the one‐tailed tests for high gamma data. One‐tailed tests were used for high gamma ERBP, as LGD effects in this band were defined as power increases.

Clusters were defined as consecutive time points for which the *t*‐statistic exceeded the threshold, and the cluster‐level statistic was computed as the sum of the *t*‐values within each cluster. The significance level (*p*‐value) of those statistics was calculated using permutation tests. To construct the permutation distribution, 10,000 random trial partitions were made and shuffled with respect to trial labels (standard vs. deviant); the cluster statistics were calculated, and the largest cluster‐level statistic was identified for each partition. Monte Carlo *p*‐values were calculated for each cluster based on the 10,000‐sample distribution set of the test statistics. To correct for multiple comparisons across recording sites, *p* values were adjusted by controlling the false discovery rate as done previously (Nourski et al. 2018a, 2018b, 2021a, 2021b). LGD effects were considered significant at *p* < 0.05. Recording sites with at least one significant LGD effect cluster were considered as exhibiting the effect.

Topography and time course of LGD effects were compared across awake, sedated, unresponsive in dexmedetomidine experiments, and awake, drowsy, and asleep in sleep experiments. Sites with significant AEP and high gamma LGD effects were plotted in Montreal Neurological Institute (MNI) coordinate space and projected onto the right hemisphere of the FreeSurfer average template brain. Left hemisphere MNI *x*‐axis coordinates were multiplied by (−1) to map them onto the right‐hemisphere common space. Regional prevalence of LGD effects was calculated for each of the four ROI groups as the percentage of recording sites exhibiting a given novelty effect (AEP LD, high gamma LD, AEP GD or high gamma GD). Comparisons of prevalence of LGD effects were performed using Fisher exact tests, with results reported as *p* values and odds ratios (OR) and their asymptotic confidence intervals. The time course of LGD effects was examined by plotting the number of sites within each ROI group exhibiting significant LGD effects as a function of time. For this analysis, data were pooled across the 7 participants of the dexmedetomidine set and the 5 participants of the sleep cohort.

## 3 Results

### 3.1 Time Course of the Experiments

Dexmedetomidine experiments were carried out in the operating room immediately prior to iEEG electrode removal. Sedation commenced with a bolus of dexmedetomidine (0.25 μg/kg), followed by infusion at a rate between 0.2 and 0.8 μg/kg/h, with subsequent increases in infusion rate to 0.8–4 μg/kg/h. All changes in infusion rate and delivery of additional bolus doses were at the discretion of the anesthesiologist. Thus, the time course of sedation and LOR during infusion of dexmedetomidine differed across participants (see Figure S2a). LOR occurred at infusion rates between 0.8 and 2 μg/kg/h in 6 out of 7 participants. Decreases in OAA/S associated with state transitions (awake to sedated and sedated to unresponsive) were paralleled by decreases in BIS values, with the only exception being a slight increase in the sedated block in participant L625 (Figure S2b).

OAA/S assessments depend on participants responding to simple verbal commands, which in turn may modulate arousal. To address this possibility, an LME model was used to compare BIS values recorded immediately before and after each of the four OAA/S assessments used to define sedated and unresponsive blocks (see Figure S1a). There was a significant (*p* = 0.00113) main effect of OAA/S assessment order (1st through 4th, i.e., before and after sedated block, and before and after unresponsive block), consistent with a decrease in BIS values over the course of the experiment. There was no significant effect based on whether BIS was measured before or after OAA/S assessment (*p* = 0.491), indicating that OAA/S assessments did not significantly affect the participants' level of arousal as measured using BIS. There was no significant interaction between the two main effects (i.e., OAA/S assessment order within the experiment and whether BIS was measured before or after OAA/S assessment; *p* = 0.976). Thus, we detected no effect of OAA/S assessment on the participants' level of arousal as measured using BIS.

Sleep experiments also had a variable time course, with experimental blocks recorded when participants were deemed asleep at 24, 53, 36, 47, and 37 min after the beginning of the session (see Figure S2c). Sleep was operationally defined by participants' lack of response to the target GD stimuli throughout the block, eyes closed, lack of response to the research team member entering the room after the block, and the presence of a peak in the alpha band (~12 Hz) indicating the presence of sleep spindles (Andrillon et al. 2011, Figure S3). Four participants stopped responding to the target stimuli during the second experimental block (“Drowsy”) within 15 min of experimental session onset. Participant L585 had a total of 6 button presses scattered throughout the last block. Notably, only one button press had timing consistent with a correct hit response to the target stimulus. The presence of these occasional button presses indicates that the participant had brief periods of arousal during the block. This block otherwise met the criteria for sleep (eyes closed, no response to the research team member and presence of an alpha peak in the iEEG data) and thus was included in the analysis. Overall, the dexmedetomidine and sleep experiments yielded comparable trajectories in the participants' decline in task performance and arousal state.

### 3.2 Exemplar Data (Dexmedetomidine)

**FIGURE 2:**
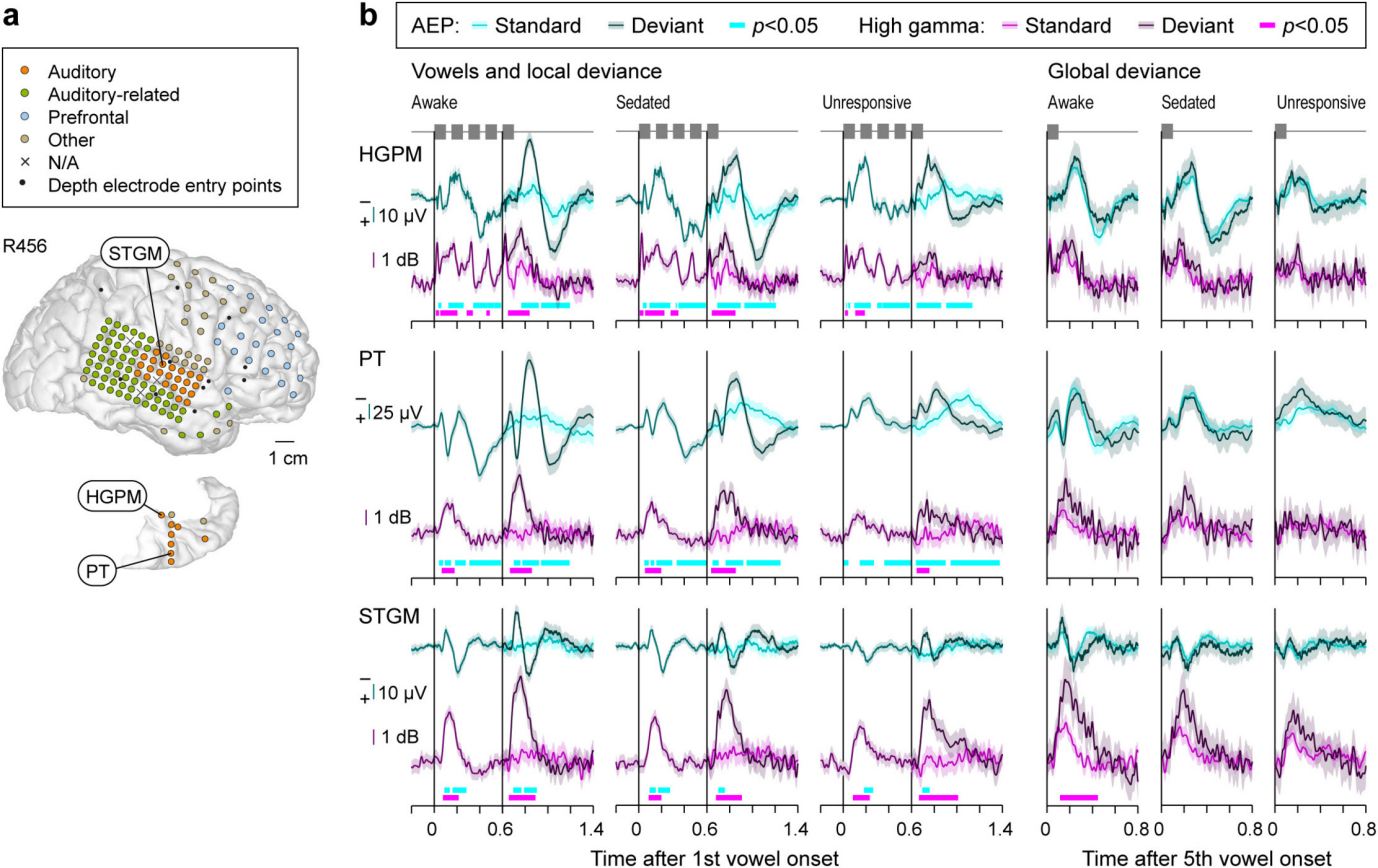
Responses to standard and deviant stimuli prior to and during induction of general anesthesia with dexmedetomidine in a representative participant (R456). (a) MRI reconstruction of the hemispheric surface and top‐down view of the superior temporal plane showing electrode coverage. Recording sites are depicted as circles, color‐coded by region‐of‐interest (ROI) group. Sites excluded from analysis due to excessive noise are denoted by “×.” Depth electrode insertion points are shown as black dots. (b) AEP waveforms (shades of cyan) and high gamma power envelopes (shades of magenta) recorded from four exemplar sites (callout boxes in (a)) in response to standard and deviant stimuli. Lines and shading represent mean values and the 95% confidence intervals, respectively. Thick lines underneath response waveforms denote statistical significance (cluster‐based permutation tests, *p* < 0.05, false discovery rate‐corrected). HGPM, Heschl's gyrus, posteromedial portion; PT, planum temporale; STGM, superior temporal gyrus, middle portion.

Prominent responses to the vowels and LGD effects were elicited in the auditory cortex on the superior temporal plane and lateral STG. This is illustrated for an exemplar participant R456 in Figure 2. Electrode coverage of the right hemispheric convexity and superior temporal plane is shown in Figure 2a (top and bottom, respectively). Figure 2b depicts AEPs and high gamma responses, recorded in the awake, sedated, and unresponsive states (left to right), for three recording sites within HGPM, PT, and STGM (top to bottom; labeled in Figure 2a). AEP and high gamma responses to the first four vowels averaged across all trials are immediately followed by separately plotted responses to the LS and LD 5th vowels (lighter and darker shading, respectively). The difference between these responses constitutes an LD effect. GD effects are illustrated in a similar manner in the right‐side panels, with responses to GS and GD 5th vowels plotted using lighter and darker shading, respectively.

Vowel stimuli elicited robust responses within all three areas of the auditory cortex, evident both as AEP complexes and high gamma ERBP (Figure 2b, left column). An envelope‐following high gamma response to the repeated vowels was evident in HGPM across all three arousal states (cf. Nourski et al. 2009). Increased responses to the 5th vowel when it differed from the first four identical vowels (LD effects) were seen as both the emergence of a biphasic AEP response and elevated high gamma power. In all three auditory cortical areas, both the vowel responses and LD effects were present in awake, sedated, and unresponsive states. Colored bars underneath response waveforms denote significant vowel responses and LD effects, as determined by cluster‐based permutation tests (cyan and magenta for AEP and high gamma, respectively). By contrast, a GD effect in this example was observed only at the STGM site in the awake state as high gamma ERBP that progressively diminished in magnitude with state change (Figure 2b, bottom row, three rightmost columns).

**FIGURE 3:**
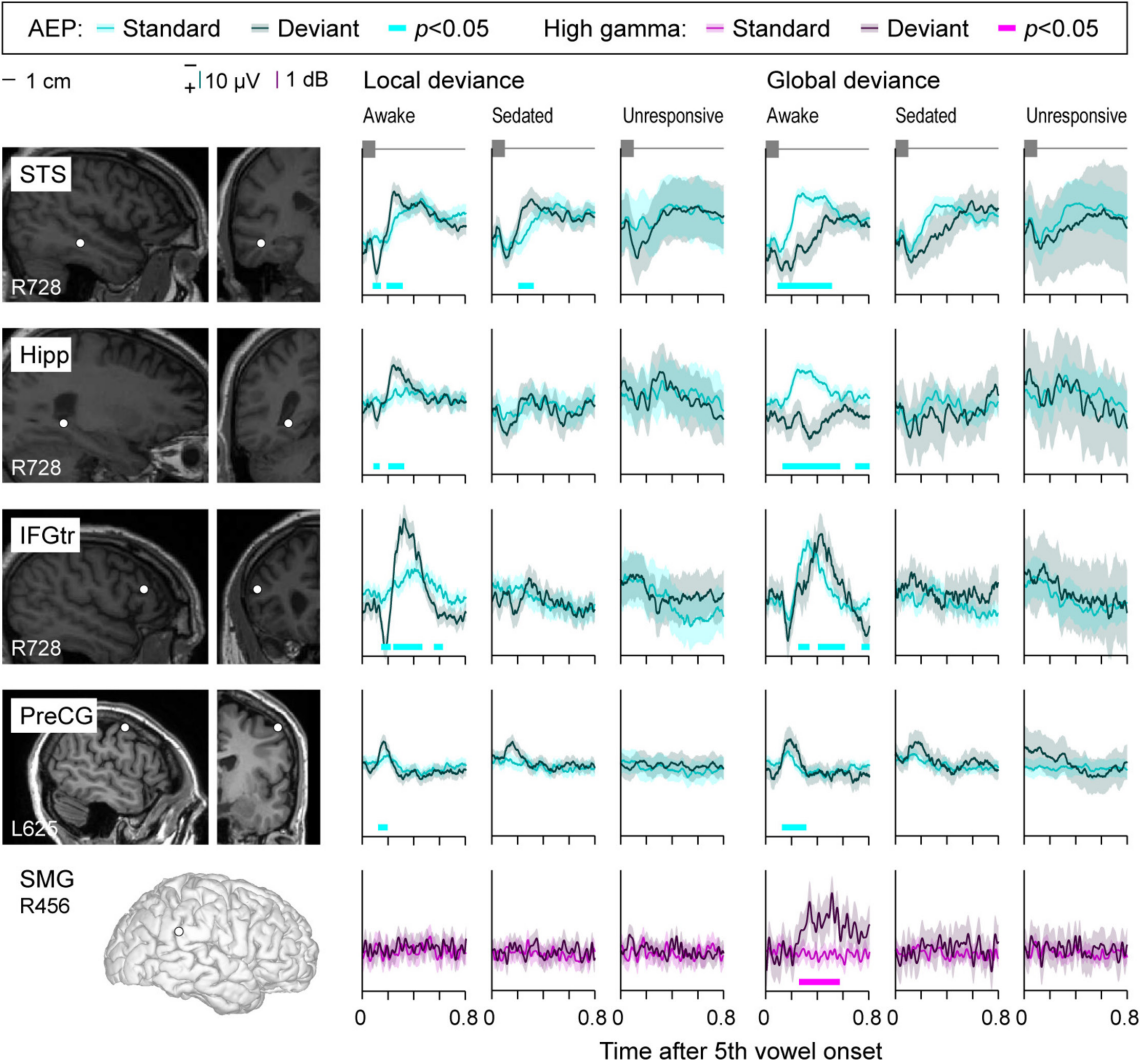
Responses to standard and deviant stimuli during dexmedetomidine experiments in representative sites outside auditory cortex. Sites are shown in the MRI sagittal and coronal views for depth electrode contacts, and on the hemispheric surface for the subdural recording contact overlying the SMG. See caption of Figure 2 for detail. Only fifth vowel responses are shown. High gamma plots in rows 1–4 and AEP plots in the bottom row are omitted as there were no significant LGD effects. STS, superior temporal sulcus; Hipp, hippocampus; IFGtr, inferior frontal gyrus, pars opercularis; PreCG, precentral gyrus; SMG, supramarginal gyrus.

LGD effects were widespread and occurred in multiple brain regions outside the auditory cortex (Figure 3). Examples are taken from different participants, as limitations in electrode coverage and sparsity of LGD effects precluded illustrating these effects in a single participant. The top four examples were obtained from depth electrodes, and their locations are shown in sagittal and coronal MRI views. The final example was obtained from a subdural electrode over the SMG. Notably, in these examples there were no significant high gamma LGD effects at sites shown in rows 1–4, and no significant AEP LGD effects at the SMG site. The latter finding was observed in the awake state at 5 sites out of 974 for the LD effect and at 17 sites for the GD effect. Overall, these exemplar responses emphasize that the AEP LD effect was more likely to be abolished during sedation at these higher processing stages compared to the auditory cortex (cf. Figure 2b) and absent in the unresponsive state. As was typical, GD effects were only observed in the awake state.

### 3.3 Topography of LGD Effects (Dexmedetomidine)

**FIGURE 4:**
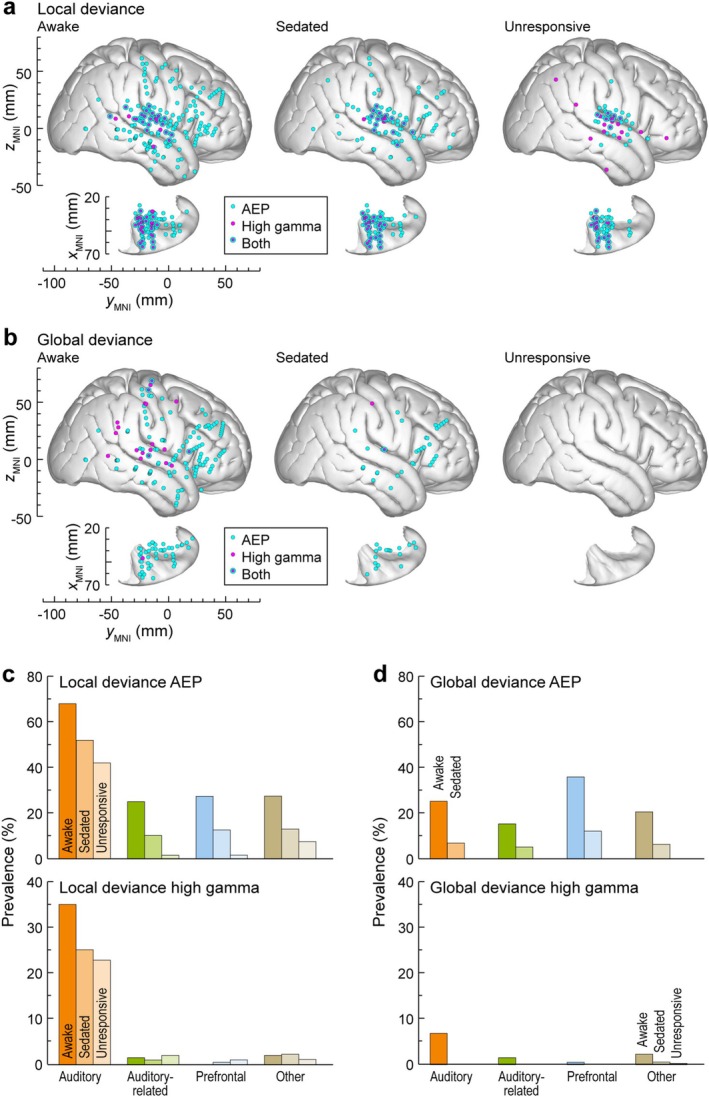
Changes in topography of LD (a) and GD effects (b) during the dexmedetomidine experiment, shown for awake, sedated and unresponsive state (columns 1–3). Summary of data from seven participants, plotted in MNI coordinate space and projected onto FreeSurfer average template brain. Left hemisphere MNI *x*‐axis coordinates (*x*_MNI_) were multiplied by (−1) to map them onto the right‐hemisphere common space. Sites with significant AEP and high gamma LGD effects are denoted by cyan and magenta symbols, respectively. (c,d) Percentages of sites across ROI groups that exhibited the LD (c) and GD (d) effects. Prevalence of AEP and high gamma LGD effects is plotted in top and bottom row, respectively. Summary of data from seven participants. Differently shaded bars represent measurements made in awake, sedated, and unresponsive states. Note that there are no bars corresponding to the unresponsive state in (d) as the AEP GD effect was completely abolished.

The spatial distribution of LGD effects in all seven participants is summarized in Figure 4 and Table 1. AEP LGD effects had a broader spatial distribution compared to high gamma (cyan and magenta symbols, respectively, in Figure 4a,b) in the awake state. Out of 863 sites in 7 participants, significant LGD effects were found in 285 (the AEP LD effect), 56 (the high gamma LD effect), 202 (the AEP GD effect), and 21 (the high gamma GD effect) sites. On the whole brain level, both the AEP LD and AEP GD effects had a higher prevalence compared to the respective high gamma effects (LD: *p* < 0.0001, OR = 7.11 [5.24, 9.65]; GD: *p* < 0.0001, OR = 12.3 [7.73, 19.4]). Sites that had both the AEP and the high gamma LD effects (*n* = 51) were mainly localized within the auditory cortex (*n* = 43), with the remaining sites in adjacent areas (2 in SMG, 5 in PostCG and 1 in InsP). Co‐occurrence of the AEP and the high gamma GD effects was limited to four sites (one in STGM, one in IFGtr and two in PreCG). Sedation and LOR led to a progressive reduction in both the AEP and high gamma LGD effects. LD effects persisted in the auditory cortex, while GD effects were completely abolished in the unresponsive state.

**TABLE 1:** Distribution of LD and GD effects across ROIs during the dexmedetomidine experiment. Data from all seven participants.

ROI group	ROI	*n*	LD						GD					
			AEP			High gamma			AEP			High gamma		
			Awake	Sedated	Unresponsive	Awake	Sedated	Unresponsive	Awake	Sedated	Unresponsive	Awake	Sedated	Unresponsive
Auditory	HGPM	35	32	28	24	20	15	11	10	2	0	1	0	0
	HGAL	5	4	3	1	2	1	0	2	2	0	0	0	0
	PP	11	10	7	5	0	0	0	8	1	0	0	0	0
	PT	18	14	15	15	6	9	8	8	3	0	0	0	0
	STGM	39	18	10	7	10	6	9	4	1	0	5	0	0
	STGP	23	11	5	3	8	2	2	1	0	0	3	0	0
Auditory‐related	MTGA	25	5	1	0	0	0	0	3	0	0	0	0	0
	MTGM	47	8	0	0	1	0	1	3	2	0	0	0	0
	MTGP	34	5	2	0	0	0	0	4	0	0	1	0	0
	STGA	6	3	2	1	0	0	0	2	1	0	0	0	0
	STS	31	21	10	2	0	1	1	14	6	0	0	0	0
	AG	16	1	1	0	0	0	1	1	0	0	0	0	0
	SMG	37	6	4	0	2	1	1	3	1	0	2	0	0
Prefrontal	ACC	13	2	1	1	0	0	0	5	4	0	0	0	0
	IFGop	14	6	5	0	0	0	0	5	0	0	0	0	0
	IFGor	6	3	1	2	0	0	0	2	0	0	0	0	0
	IFGtr	43	17	10	0	0	1	1	19	11	0	1	0	0
	MFG	52	12	4	0	0	0	0	19	8	0	0	0	0
	OG	42	9	3	0	0	0	1	12	0	0	0	0	0
	SFG	20	3	0	0	0	0	0	6	0	0	0	0	0
Other	Amyg	23	5	1	0	0	0	0	5	1	0	0	0	0
	Hipp	29	13	2	0	0	0	0	8	1	0	0	0	0
	PHG	8	1	0	0	0	0	0	1	0	0	0	0	0
	ParaCL	2	0	0	0	0	0	0	0	0	0	0	0	0
	PostCG	39	20	16	14	5	6	4	8	3	0	3	2	0
	PreCG	69	19	9	4	0	1	0	11	1	0	4	0	0
	Caud	7	6	0	0	0	0	0	0	0	0	0	0	0
	CingMA	8	1	0	0	0	0	0	3	3	0	0	0	0
	CingMP	6	1	0	0	0	0	0	0	0	0	0	0	0
	CingPD	4	0	0	0	0	0	0	0	0	0	0	0	0
	CingPV	1	0	0	0	0	0	0	1	0	0	0	0	0
	FG	15	2	0	0	0	0	0	3	0	0	0	0	1
	GR	10	1	0	1	0	0	0	2	0	0	0	0	0
	IPS	3	0	0	0	0	0	0	0	0	0	1	0	0
	ITG	17	1	0	0	0	0	0	1	0	0	0	0	0
	InsA	17	5	4	1	0	0	0	9	5	0	0	0	0
	InsP	19	13	11	5	2	1	0	10	6	0	0	0	0
	LingG	4	1	0	0	0	0	0	0	1	0	0	0	0
	MOG	2	0	0	0	0	0	0	0	0	0	0	0	0
	PMC	24	2	2	0	0	0	0	1	1	0	0	0	0
	PreCun	3	0	0	0	0	0	0	1	0	0	0	0	0
	SubCG	2	0	0	1	0	0	0	0	0	0	0	0	0
	TP	28	4	0	0	0	0	0	5	0	0	0	0	0
	FOp	2	0	0	0	0	0	0	0	0	0	0	0	0
	POp	2	0	0	0	0	0	0	2	0	0	0	0	0

Prevalence of AEP LGD effects is shown in Figure 4c,d, respectively. The AEP LD effect was attenuated throughout the brain with sedation and became essentially confined to the auditory cortex following LOR (see Figure 4c). The few sites outside of the auditory cortex that maintained a significant AEP LD effect in the unresponsive state were mostly located in the perisylvian sensorimotor cortex (PostCG, PreCG) and InsP (Figure 4a). High gamma LGD effects underwent a marked decrease in prevalence outside of the auditory cortex in the sedated and unresponsive states. The AEP LD effect within the auditory cortex (including HGPM, other ROIs within the superior temporal plane, and the lateral STG) was more resistant to sedation than the GD effect (*p* = 0.0120, OR = 0.357 [0.160, 0.796]; see Figure 4c,d). By contrast, this difference in sensitivity to sedation between AEP LD and GD effects was not observed within the auditory‐related and prefrontal cortex (auditory‐related: *p* = 0.824, OR = 0.817 [0.337, 1.98]; prefrontal: *p* = 0.392, OR = 0.733 [0.373, 1.44]).

Consistent with previous findings (Nourski et al. 2018b, 2021a), the prevalence of GD effects, but not LD effects, was related to task performance. This is illustrated in Figure S4 for AEP LGD effects. By contrast, a significant AEP GD effect in the awake state was predominantly seen in participants who exhibited better task performance (symbols plotted in warmer colors). In the sedated state, the AEP GD effect was present only in the four participants who still performed the task (hit rate between 29.2% in R728 and 65.1% in R458). This was not the case for the AEP LD effect, which was present in good and poor performers alike in all three states.

LGD effects were not uniformly distributed within the auditory‐related cortex (see Table 1). The STS had a relatively high prevalence of the AEP LD effect (67.7% sites in the awake state). While there was a decrease with sedation (31.5%), the AEP LD effect remained more prominent in the STS compared to other auditory‐related ROIs. However, in contrast to the auditory cortex, it was essentially abolished following LOR (2 sites or 6.45%). The potential importance of the STS in the processing of auditory novelty is further emphasized by the relative persistence of the AEP GD effect during sedation. Once again, the prevalence of the AEP GD effect in the STS (45.2%) was higher than in any other auditory‐related ROIs. The second ROI with relatively prominent LGD effects was the SMG. The AEP LD effect was found in the SMG in the awake (16.2% sites) and sedated states (10.8%) and was absent in the unresponsive state. Thus, unlike in the canonical auditory cortex, the AEP LD effect in the auditory‐related cortex (particularly, STS and SMG) exhibited high sensitivity to LOR induced by dexmedetomidine.

Within prefrontal cortex, all three subdivisions of the IFG (IFGop, IFGtr, and IFGor) featured the AEP LD effect in the awake state and, to a lesser extent, in the sedated state. The AEP GD effect in IFGtr and MFG was preserved in the sedated state in 3 out of 7 participants (R458, L625 and R720) who had above‐average GD target detection task performance in the sedated state (see Figure S4). Within sensorimotor cortex, PostCG had a higher prevalence of LGD effects compared to PreCG in the awake state (AEP LD: 51.3% sites and 27.5%, respectively; high gamma LD: 12.8% vs. 0%; AEP GD: 20.5% vs. 15.9%; high gamma GD: 7.69% vs. 5.80%). LD effects were partially preserved following LOR, mainly at sites clustered around the Sylvian fissure. The AEP LD effect within limbic areas (amygdala, hippocampus, and PHG) primarily occurred in the hippocampus. The AEP GD effect was more evenly distributed between hippocampus and amygdala (27.6% and 21.7% of recording sites in the awake state, respectively) and were particularly sensitive to sedation (4.35% and 3.45% in the sedated state, respectively). LGD effects were differentially distributed between InsP and InsA, with LD effects focused in InsP and GD effects more prevalent in InsA. Novelty responses in InsP were resistant to dexmedetomidine. Of special note, the AEP GD effect was also found in CingMA, consistent with InsA and CingMA being major foci of the salience network (Menon and Uddin 2010). Additionally, 6 out of 7 sites in the caudate nucleus exhibited the AEP LD effect, which was only present in the awake state.

The time course of LGD effects relative to the fifth vowel onset is summarized in Figure S5. Key features in the time course of LD and GD effects include a longer latency of GD effects compared to LD and a marked temporal overlap in regional representation of auditory novelty, with concomitant LGD effects occurring in multiple brain regions. These findings in the awake state are consistent with those reported in a previously studied participant cohort (Nourski et al. 2018a, 2018b). In the sedated state, a bimodal distribution in the timing of the AEP LD effect emerged, with two peaks at ~200 and 450 ms. A bimodal temporal distribution was also observed for the AEP GD effect in the awake state, primarily driven by areas outside canonical auditory cortex. Overall, the analysis depicted in Figure S5 highlights the differential sensitivity to brain state change along the auditory cortical hierarchy, complementing the topographic representation depicted in Figure 4.

### 3.4 Exemplar Data (Sleep)

**FIGURE 5:**
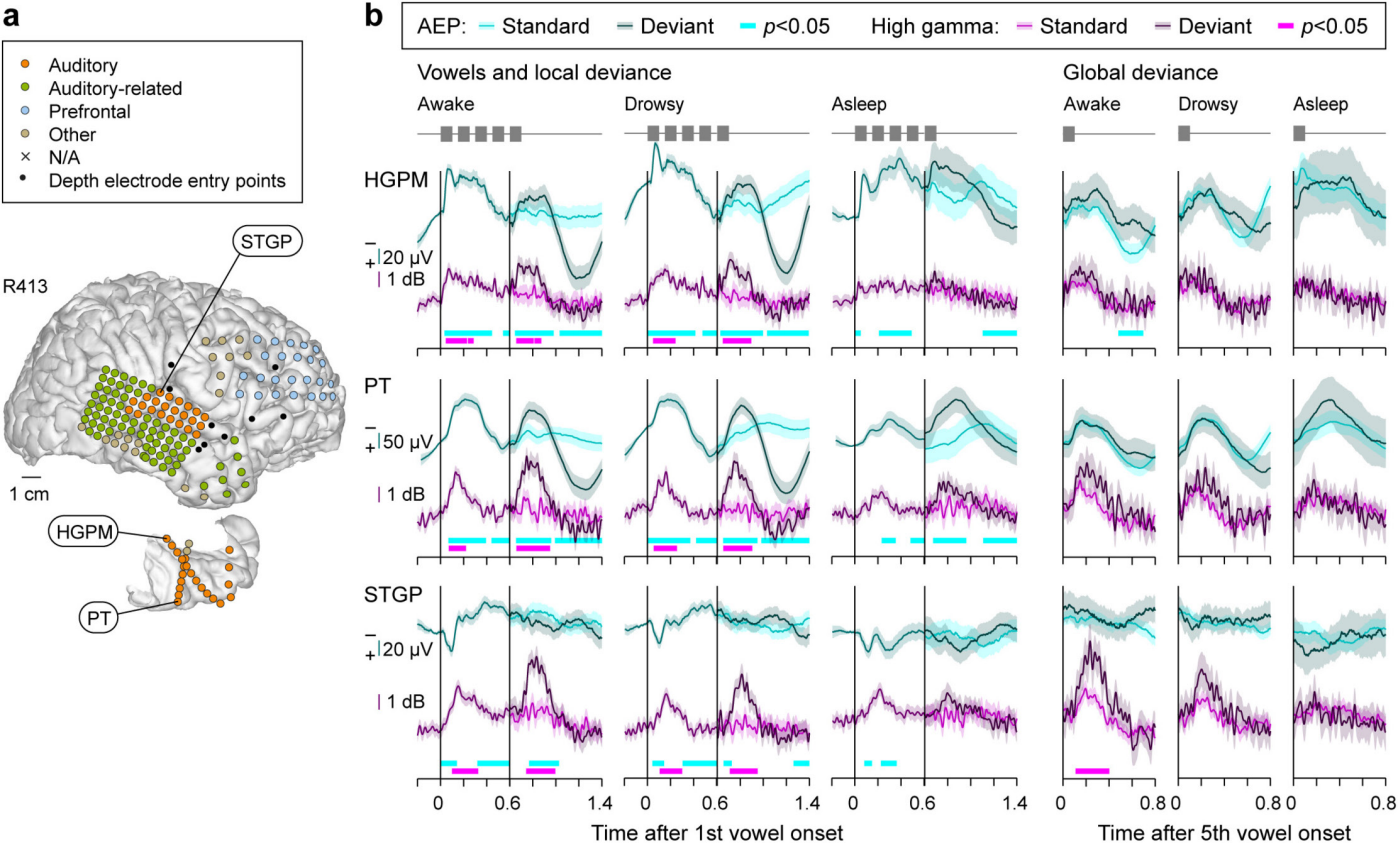
Responses to standard and deviant stimuli during a sleep experiment in a representative participant (R413). See caption of Figure 2 for details.

Changes in LGD effects upon transition from wakefulness to sleep paralleled those during the dexmedetomidine experiment. Figure 5a shows electrode coverage in participant R413, with three auditory cortical sites used to illustrate typical patterns found within HGPM, PT, and STGP in Figure 5b. The second stimulus block, during which the participant did not press the response button to target GD stimuli, was defined as the drowsy condition. Increased alpha power, a sign of sleep spindles and stage 2 sleep, was not present during this second block (see Figure S3). AEP responses to vowels and the AEP LD effect in HGPM and PT were present in all three states. The high gamma LD effect, while present in awake and drowsy states, was abolished during sleep. In this example, GD effects were present in the AEP only in the awake state within HGPM. The STGP site maintained a significant AEP response to vowels across the three brain states, but the AEP LD effect was lost during sleep. The AEP GD effect was not noted in the AEP at this site. There was a reduction of high gamma responses to vowels below the significance threshold and a loss of the high gamma LD effect during sleep. The high gamma GD effect decreased below the significance threshold during drowsiness and was abolished during sleep.

**FIGURE 6:**
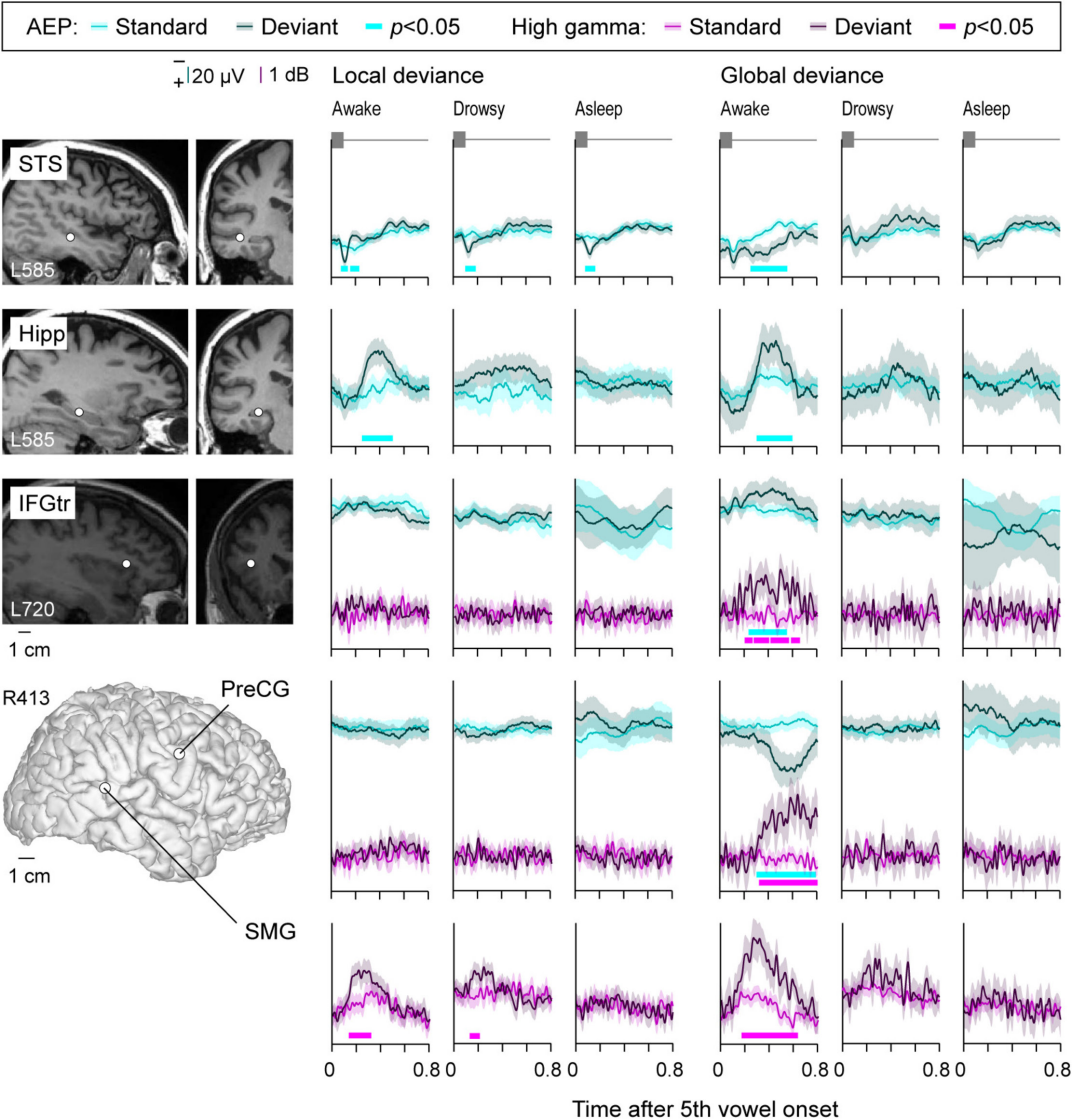
Responses to standard and deviant stimuli during sleep experiments in representative sites outside auditory cortex (top to bottom: superior temporal sulcus [STS], hippocampus [Hipp], inferior temporal gyrus pars triangularis [IFGtr], precentral gyrus [PreCG], supramarginal gyrus [SMG]). See caption of Figure 3 for details.

Examples of LD and GD effects observed in brain regions outside auditory cortex are shown in Figure 6. A short latency AEP LD effect was observed in STS and was present in all three states. The AEP GD effect seen at the same site had a longer onset latency and was only present while the participant was awake and performing the task. Within the hippocampus, both AEP LD and GD effects were restricted to the awake state. GD effects could be observed in the absence of LD effects (as seen in IFGtr and PreCG examples in Figure 6). Finally, while it was more generally common for novelty effects to occur in the AEP signal, LD and GD effects sometimes occurred in the high gamma band only, without concomitant AEP novelty effects (SMG site in Figure 6; cf. SMG site in Figure 3). This was observed in 5 sites out of 764 for the LD effect and in 17 sites for the GD effect. Overall, the examples shown in Figures 5 and 6 highlight the similarities between effects of dexmedetomidine and natural drowsiness/sleep (cf. Figures 2 and 3), particularly with respect to differential sensitivity of LD and GD effects to change in brain state.

### 3.5 Topography of LGD Effects (Sleep)

**FIGURE 7:**
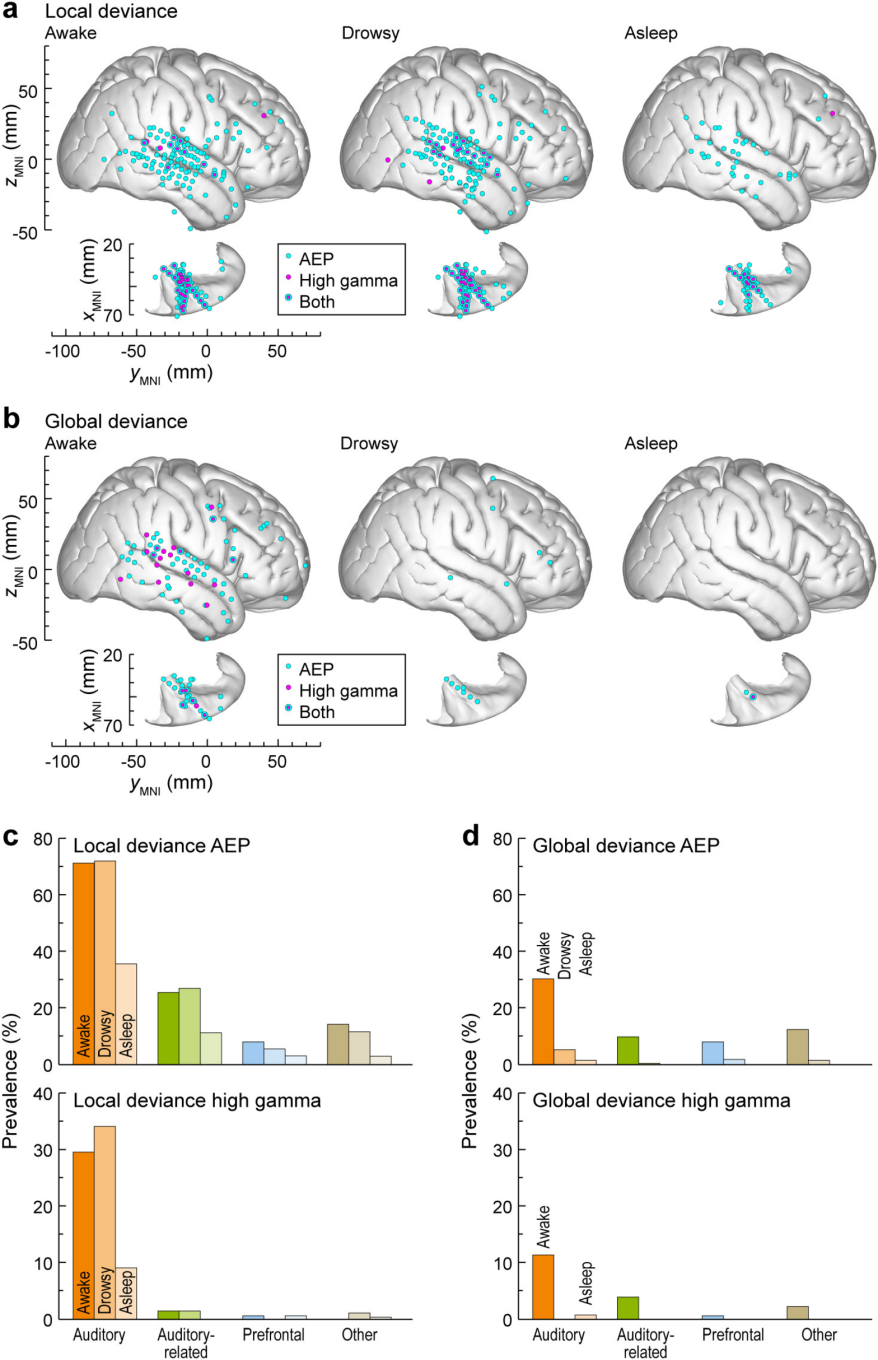
Changes in topography and prevalence of LD and GD effects during the sleep experiment, shown for awake, drowsy, and asleep state (columns 1–3). Summary of data from five participants. See caption of Figure 4 for details.

Changes in LGD effects during daytime sleep were similar to those observed following LOR during dexmedetomidine experiments (Figure 7, Table 2). As in the dexmedetomidine experiments, on the whole brain level, both the AEP LD and the AEP GD effects were more common than the respective high gamma effects (LD: *p* < 0.0001, OR = 5.83 [4.11, 8.25]; GD: *p* < 0.0001, OR = 3.94 [2.59, 5.99]). The AEP LD effect in the auditory and auditory‐related cortex was unaffected by drowsiness and was relatively spared during sleep, while the AEP GD effect was highly sensitive to brain state change. While there was a prominent decrease in the prevalence of the AEP LD effect by ~50%, many sites in these regions still responded to LD.

**TABLE 2:** Distribution of LD and GD effects across ROIs during the sleep experiment. Data from all five participants.

ROI group	ROI	*n*	LD						GD					
			AEP			High gamma			AEP			High gamma		
			Awake	Drowsy	Asleep	Awake	Drowsy	Asleep	Awake	Drowsy	Asleep	Awake	Drowsy	Asleep
Auditory	HGPM	23	22	23	20	18	18	8	12	4	0	3	0	0
	HGAL	13	13	12	9	5	5	2	6	3	2	2	0	1
	PP	14	1	4	1	0	0	0	2	0	0	0	0	0
	PT	22	18	18	9	8	7	2	3	0	0	2	0	0
	STGM	16	10	11	1	1	5	0	10	0	0	1	0	0
	STGP	44	30	27	7	7	10	0	7	0	0	7	0	0
Auditory‐related	MTGA	7	1	1	2	0	0	0	0	0	0	0	0	0
	MTGM	34	9	16	3	0	0	0	3	0	0	1	0	0
	MTGP	57	17	5	2	0	1	0	5	0	0	1	0	0
	STGA	10	3	2	1	1	1	0	1	0	0	1	0	0
	STS	33	12	14	7	1	0	0	4	1	0	2	0	0
	AG	25	1	5	1	0	0	0	2	0	0	1	0	0
	SMG	38	9	12	7	1	1	0	5	0	0	2	0	0
Prefrontal	ACC	7	0	0	0	0	0	0	0	0	0	0	0	0
	FP	14	0	0	0	0	0	0	2	0	0	0	0	0
	IFGop	13	3	2	0	0	0	0	1	0	0	0	0	0
	IFGor	6	0	0	0	0	0	0	0	0	0	0	0	0
	IFGtr	33	3	2	0	0	0	0	3	2	0	1	0	0
	MFG	48	3	3	2	1	0	1	6	0	0	0	0	0
	OG	37	4	2	3	0	0	0	1	1	0	0	0	0
	SFG	4	0	0	0	0	0	0	0	0	0	0	0	0
Other	Amyg	10	2	0	0	0	0	0	2	0	0	1	0	0
	Hipp	13	4	1	0	0	0	0	3	0	0	1	0	0
	PHG	9	0	0	0	0	0	0	0	0	0	0	0	0
	PostCG	21	6	5	2	0	0	0	3	1	0	0	0	0
	PreCG	38	10	9	2	0	0	1	7	1	0	1	0	0
	CingMA	5	0	0	0	0	0	0	1	0	0	1	0	0
	CingMP	1	0	0	0	0	0	0	0	0	0	0	0	0
	FG	19	1	1	0	0	0	0	0	0	0	0	0	0
	GR	10	1	1	0	0	0	0	4	0	0	0	0	0
	ITG	40	5	3	0	0	1	0	3	0	0	0	0	0
	InsA	10	2	0	1	0	0	0	0	0	0	0	0	0
	InsP	25	3	5	2	0	0	0	4	0	0	0	0	0
	LingG	4	0	0	0	0	0	0	0	0	0	0	0	0
	MOG	1	0	0	0	0	0	0	0	0	0	0	0	0
	PMC	20	1	0	0	0	0	0	1	2	0	1	0	0
	Precun	2	0	0	0	0	0	0	0	0	0	1	0	0
	SubCG	2	0	0	1	0	0	0	1	0	0	0	0	0
	SubInn	3	0	0	0	0	0	0	0	0	0	0	0	0
	TP	26	2	3	0	0	0	0	2	0	0	0	0	0
	FOp	1	0	0	0	0	1	0	0	0	0	0	0	0
	POp	6	1	3	0	0	1	0	2	0	0	0	0	0

A significant AEP GD effect in the awake state was only detected in three participants out of five who had exhibited better task performance (R413: 50.6% hit rate, L585: 47.2%, R720 77.8%), but not in the two who performed poorly (L372: 12.5%, L514: 5.56%) (Figure S6). In the drowsy state, the AEP GD effect was only observed in one participant (L585). This participant was the only one out of five who still performed the task while drowsy (see Figure S2). Likewise, the two sites in HGAL that exhibited a significant AEP GD effect in the asleep state were found in the same participant. This further highlights the strong relationship between physiology and behavior for detecting and responding to GD, consistent with dexmedetomidine data (cf. Figure S4).

A notable difference between dexmedetomidine and sleep experiment results was that in the latter experiment, there was no decrease in the AEP LD effect prevalence with drowsiness in auditory and auditory‐related cortex (see Figure 7a,c, Table 2). Indeed, several ROIs exhibited an increase in the number of sites with a significant AEP LD effect in the drowsy state. This observation suggests that natural drowsiness and drug‐induced sedation, while both intermediate states between wakefulness and (presumed) unconsciousness, are not equivalent with respect to LD processing.

A notable difference between dexmedetomidine and sleep experiment results was that in the latter experiment there was no decrease in the AEP LD effect prevalence with drowsiness in the auditory and auditory‐related cortex (see Figure 7a,c, Table 2). It should be stressed that the awake states in the dexmedetomidine and sleep experiment were likely non‐equivalent given the differences in the level of arousal in part due to the experimental environment. Namely, dexmedetomidine experiments were conducted in the brightly lit operating room in the morning with the participants lying on the gurney, while sleep experiments were carried out in the dimly lit monitoring suite in the afternoon, with the participants comfortably lying in their bed. Additionally, there was a marked decline in the AEP LD effect prevalence between the drowsy and sleep states, especially in STGM. This further highlights the role of more caudal auditory areas, including PT and STGP, in monitoring the acoustic environment following LOR during general anesthesia and sleep.

The time course of LGD effects relative to the fifth vowel onset is summarized in Figure S7. Transition from awake to drowsy and asleep states was characterized by a progressive emergence of a bimodal distribution of the AEP LD effect, similar to that seen during dexmedetomidine sedation and unresponsiveness. (Figure S4a, cf. Figure S3a). The high gamma LD effect was primarily restricted to auditory cortex and occurred within 400 ms, consistent with the timing observed in the dexmedetomidine experiment. GD effects had a slower time course, reaching significance ~200 ms later than LD effects. Additionally, there was a difference in the time course of the AEP GD effect between awake states in the dexmedetomidine and sleep experiments. The latter had a slower onset and reaching its first peak at around 500 ms following the 5th vowel onset, while in the pre‐dexmedetomidine awake condition the initial prevalence peak occurred at around 300 ms. The discrepancies in the timing of LGD effects between the two experiments may reflect differences in the level of arousal associated with experimental environment and participants' task performance.

### 3.6 Emergence of LD Effects During Dexmedetomidine Unresponsiveness and Sleep

In both the dexmedetomidine and sleep experiments, a small minority of sites exhibited significant AEP or high gamma LD effect in the unresponsive and sleep states, without corresponding effects during wakefulness. Examples of such sites are presented in Figures S8 and S9 for the dexmedetomidine and sleep experiment, respectively. In these figures, examples of sites that did exhibit LD effects in the awake state are also presented for comparison. Examination of LD effects on a site‐by‐site basis suggested that in some cases there was no evidence for an LD effect in the awake state, yet it appeared in states of reduced arousal (e.g., sites HGPM (2), PT, STGM in Figure S8 and sites HGPM (2) and SMG (2) in Figure S9). In other cases, however, the appearance of LD effects could be interpreted as false positives (e.g., STS site in Figure S9). Other sites in the same participants did exhibit LD in the awake state (e.g., sites HGPM (1), HGAL, STGP in Figure S8 and sites HGPM (1), STGP and SMG (1) in Figure S9).

In the dexmedetomidine experiment, 13 of 974 sites (1.33%) had a significant AEP LD effect in the unresponsive state in the absence of a significant effect in the awake state (cf. 285 sites [29.3%] in the awake state). Four of these sites were located in the auditory cortex (HGPM, PT, STGM), three in prefrontal ROIs (IFGor, ACC), and the remaining six were in PostCG, InsA, InsP, SubCG, and GR. Likewise, 13 sites (1.33%) had a significant high gamma LD effect in the unresponsive state in the absence of a significant effect in the awake state (cf. 56 sites [5.75%] in the awake state). Seven of these sites were located in the auditory cortex (HGPM, PT, STGM), four in auditory‐related cortex (AGA, MTGM, SMG, STS), and two in prefrontal cortex (IFGtr, OG).

In the sleep experiment, 18 of 764 sites (2.36%) had a significant AEP LD effect in the unresponsive state in the absence of the corresponding effect in the awake state (cf. 197 sites [25.8%] in the awake state). Four of these sites were located in the auditory cortex (HGPM, PT, STGP), 10 in auditory‐related areas (STS, MTGM, MTGA, SMG, AGA), three in prefrontal ROIs (MFG, OG), and one in SubCG. Three sites (0.393%) had a significant high gamma LD effect in the asleep state in the absence of a significant effect in the awake state (cf. 43 sites [5.63%] in the awake state). The three sites were located in HGAL, MFG, and PreCG. Thus, sites with significant LD effects in the unresponsive/asleep, but not awake state, were scattered across multiple levels of the cortical auditory processing hierarchy.

## 4 Discussion

This is the first published report of iEEG responses to auditory stimulation following administration of dexmedetomidine. The goals of the present study were twofold. First, we sought to determine whether previously reported effects of propofol on auditory novelty responses could be reproduced by an anesthetic drug with a different mechanism of action. Propofol modulates GABAergic inhibition, while dexmedetomidine is a selective alpha‐2 adrenergic agonist. These differences might lead to markedly different LGD responses during sedation and LOR. Furthermore, dexmedetomidine sedation and anesthesia have been compared to natural non‐rapid eye movement sleep (Akeju et al. 2018; Lee 2019; Moody et al. 2021). This comparison is supported by several lines of evidence. As in sleep, patients are often verbally arousable during dexmedetomidine administration (Lee 2019). Polysomnographic staging of data recorded during dexmedetomidine anesthesia yields typical sleep EEG activity patterns, including spindles (Huupponen et al. 2008; Akeju et al. 2018; Moody et al. 2021). Finally, dexmedetomidine acts on endogenous sleep/arousal circuits and causes a sleep‐like decrease in norepinephrine release by suppressing neural activity in the locus coeruleus (Correa‐Sales et al. 1992; Nelson et al. 2003). Therefore, a secondary goal of the study was to compare changes in auditory predictive processing during dexmedetomidine administration and natural sleep.

### 4.1 Modulation of Auditory Novelty Processing: Comparison of Propofol and Dexmedetomidine Results

Effects of dexmedetomidine are similar to those previously reported for propofol using the same experimental paradigm (Nourski et al. 2018b). For both anesthetics, sedation led to a reduction in the prevalence of LD effects. This reduction was particularly prominent within the prefrontal cortex. Another similarity was the finding that LOR was associated with a near‐complete loss of the AEP LD effect in auditory‐related and prefrontal cortex, and confinement of the AEP LD effect to auditory cortex. The effect on the processing of LD was paralleled by loss of GD effects throughout the brain, including auditory cortex. Notably, the presence of GD, but not LD, effects was related to participants' engagement in the target detection in both studies. When participants stopped responding to the target GD stimuli, GD effects dissipated.

Responses to global novelty are strongly task‐dependent even in the awake state and can be attenuated when attentional focus is shifted from the auditory GD stimuli to a visual task (Kompus et al. 2020; Nourski et al. 2021a). This observation emphasizes that auditory GD processing indexes attention and effort rather than consciousness per se. Even though the absence of GD in a laboratory setting may be a marker of LOC following wakefulness with an active task, the sensitivity of GD effects to task performance limits their clinical utility in the operating room and intensive care units. A corollary of this premise is that the absence of GD effects in paradigms meant to assess the brain state of behaviorally unresponsive patients cannot be used as a marker for LOC. A more subtle physiological finding, such as the restriction of LD effects to auditory cortex, might serve as a more appropriate marker for this state.

Previous studies examining the effects of dexmedetomidine on auditory processing relied on a variety of different experimental paradigms, thereby making generalizations across studies challenging. Using scalp‐recorded EEG, Kallionpää et al. (2018) found that both dexmedetomidine and propofol attenuated the difference between responses to congruous vs. incongruous words at the end of a sentence (“the N400 effect”). This effect bears similarity to the GD effect measured in the current study, as both depend on integration of information over an extended period of time (seconds) for deviance to be detected. Kallionpää et al. (2018) found a difference between effects of dexmedetomidine and propofol, wherein a scalp‐recorded negativity in the 400 ms range was present with dexmedetomidine but absent with propofol. However, this negativity did not distinguish between congruent and incongruent words, indicating that it may reflect a process distinct from the N400. Thus, it is problematic to relate this observation to changes in the AEP GD effect described in the present study and that of Nourski et al. (2018b).

Frölich et al. (2017) compared the extent of auditory cortical activation measured by fMRI while participants passively listened to multiple snippets of music during moderate degrees of sedation with either dexmedetomidine, midazolam, or propofol. They found a decrease in activation with dexmedetomidine and midazolam, but not propofol. While an explanation for this curious result was not provided, an intracranial study found preserved early AEP components and enhanced phase‐locking to click trains within HGPM under propofol anesthesia (Nourski et al. 2017). By contrast, the auditory cortex surrounding HGPM showed reduced AEPs and high gamma responses. One plausible explanation for the differences in the effects of drugs reported by Frölich et al. (2017) is that propofol may decrease inhibitory feedback control to a greater extent than either dexmedetomidine or midazolam.

Supporting this idea are functional connectivity data that showed a greater degree of disruption in auditory cortical connectivity with propofol compared to dexmedetomidine and stage 3 sleep (Figure 1B in Guldenmund et al. 2017). By contrast, the study found that thalamic inputs into sensory cortices (including auditory) were relatively unaffected by the two drugs. This sparing can account for the presence of neural responses at early auditory cortical levels under general anesthesia (cf. Nourski et al. 2017, 2021b). Indeed, the persistence of the AEP LD effect in the auditory cortex under general anesthesia can be explained by stimulus‐specific adaptation, which persists within early auditory cortex in both awake and anesthetized states (Brosch and Schreiner 1997; Ulanovsky et al. 2003; Fishman and Steinschneider 2012).

### 4.2 Sleep

Changes in auditory novelty responses during sleep as found in the present study are generally consistent with previous results obtained using simultaneous EEG/MEG recordings (Strauss et al. 2015). Similarities include loss of GD effects and disruption of LD effects outside auditory cortex. The persistence of LD effects in auditory cortex during sleep is consistent with preservation of single neuron and high gamma auditory cortical activity during sleep reported by Hayat et al. (2022). These findings indicate that lower sensory areas continue to monitor the auditory environment for potentially behaviorally relevant stimuli (Strauss et al. 2015).

Changes in LGD effects during sleep share multiple characteristics with those occurring during the induction of general anesthesia with both dexmedetomidine and propofol. In all cases, GD effects were highly sensitive to changes in brain state. As with anesthetic‐induced LOR, the extent of LD effects during sleep was largely restricted to canonical auditory cortex. The similarities in the effects on cortical auditory novelty responses observed with propofol, dexmedetomidine, and sleep indicate that these effects are reflective of general changes in the level of arousal. Thus, current data support the generalizability of changes in cortical auditory novelty detection to different general anesthetics and natural sleep. Additional study using other anesthetics such as volatile gases (e.g., isoflurane) will be required to validate this prediction.

An interesting finding specific to sleep was a pronounced reduction in LD effects within STGM that was not observed during LOR due to dexmedetomidine. One might expect the opposite relationship given that natural sleep is an arousable state, while the unresponsive state during the dexmedetomidine experiment is not (by definition). It should be noted that this change was driven by two participants out of five (L372 and R413) who had extensive electrode coverage of STGM. Loss of LD effect in STGM during sleep observed in the present study is reminiscent of the loss of a specific higher‐order mismatch negativity peak reflecting prediction error during sleep reported by Strauss et al. (2015). Additional studies will be necessary to confirm or refute whether this difference can be generalized beyond the participant cohorts studied here. Overall, the similarities between dexmedetomidine (*N* = 7) and sleep (*N* = 5) in the present study may be more meaningful than their differences. The latter may be in part attributed to relatively small sample sizes, sampling bias due to different participant cohorts with differences in electrode coverage, task performance, and experiment time course across participants.

### 4.3 LGD Effects Across Cortical Networks

AEP LGD effects had a higher prevalence and a broader spatial distribution compared to high gamma, consistent with previous iEEG LGD reports (Nourski et al. 2018a, 2018b, 2021a). The broader extent of AEPs likely reflects postsynaptic activity driven by lower‐level regions exhibiting gamma activity (Pigorini et al. 2024). Of note, a minority of sites exhibited significant high gamma LGD effects in the absence of significant AEP effects. This finding is likely secondary to a combination of methodological and physiological factors. The former—statistical significance thresholds—can arise as the stimulus paradigm (auditory novelty) requires that the deviant stimuli be relatively rare. This yields a relatively low trial count and thus can negatively affect the signal‐to‐noise ratio of the averaged data. This can lead to a false negative outcome of a statistical test, particularly for low‐amplitude AEP deviant responses. Physiological factors include those related to the summation of postsynaptic potentials and variability in AEP waveform morphologies within anatomical regions (Howard et al. 2000; Steinschneider et al. 2011).

In the current study, many ROIs outside the canonical auditory cortex exhibited AEP LGD effects. One ROI of note is the hippocampus, which displayed a relatively high prevalence of AEP LGD effects. This finding supports the interpretation that the hippocampus contributes to auditory novelty detection. It is consistent with its role as a generator of scalp‐recorded P3b (Halgren et al. 1995), a physiologic novelty response related to the AEP GD effect (Kok 2001). This finding is also consistent with previous work highlighting engagement of cortico‐limbic circuits in novelty detection that facilitates subsequent memory for novel events (Knight and Nakada 1998; Strauss et al. 2015). The AEP GD effect was observed in InsA and CingMA, implicating engagement of the salience network in the processing of global novelty (Menon and Uddin 2010; Blenkmann et al. 2019). Prominent LGD effects were seen in PreCG and PostCG during wakefulness and, to a lesser degree, in states of reduced arousal. These results are in agreement with recent work that has emphasized the role of sensorimotor cortex in auditory processing (Shtyrov et al. 2014; Cheung et al. 2016). Later onset of the LGD effects in sensorimotor cortex compared to auditory cortical ROIs, as well as the presence of these effects in dorsal PreCG and PostCG, argues against the interpretation that these novelty responses represent volume‐conducted activity from subjacent auditory ROIs. Finally, the AEP LD effect was observed in the caudate nucleus, consistent with its reported contribution to novelty detection (Sanjuan et al. 2018). Taken together, these findings indicate the involvement of multiple cortical networks beyond those subserving auditory sensory processing.

### 4.4 Emergence of LD Effects During Dexmedetomidine Unresponsiveness and Sleep

In both the dexmedetomidine and sleep experiments, a small minority of sites exhibited significant LD effects in states of reduced arousal without corresponding effects in the awake state. This intriguing finding could in some cases be attributed to occasional false positive results of cluster‐based permutation tests, yet in others it appeared to reliably represent an emergence of novelty effects in states of reduced arousal. General anesthesia can “unmask” responses subserved by circuits otherwise suppressed in the awake state. A previous study reported an enhancement of phase‐locked responses to click trains in HGPM in the unresponsive state following induction of general anesthesia with propofol (Nourski et al. 2017). This enhancement was attributed to suppression of top‐down modulatory signaling with sparing of thalamocortical afferent activity.

### 4.5 Caveats and Limitations

The relatively small cohort sizes examined here and in our previous relevant work (Nourski et al. 2018b) warrant caution when interpreting the results in the context of neural correlates of auditory perception. In general, similarities between effects of propofol, dexmedetomidine, and sleep on auditory novelty responses are more meaningful than differences between the three experiments, as the differences may in part be attributed to differences in electrode coverage between participant cohorts or driven by individual participants' data.

In the present study, some areas, particularly in parietal and especially occipital cortex, were less densely sampled than those in temporal and frontal cortex. This limitation was secondary to clinical indications for electrode implantation which targeted likely epileptic seizure foci. It is envisioned that temporal, parietal, and occipital areas (“the back of the brain”) play a direct role in specifying the content of consciousness, contrasting theories of consciousness that posit a more prominent role for frontal areas (“the front of the brain”) (Boly et al. 2017). Consequently, the present study is not well‐positioned to contribute to this “front vs. back” debate regarding the neural correlates of consciousness. Instead, this study focused on the auditory sensory modality, taking advantage of extensive electrode coverage of temporal cortex. It remains to be determined whether the effects of dexmedetomidine and sleep on conscious auditory perception can be generalized to other sensory modalities, such as visual and somatosensory.

The participants in this study were epilepsy patients and thus may not be entirely representative of the general population. However, results of the present study were consistent across multiple participants with different neurologic histories, antiseizure medication regimens, and seizure focus locations. Additionally, the findings were in agreement with previous work carried out in a different participant cohort (Nourski et al. 2018b). All participants successfully engaged in multiple additional experimental protocols beyond those described in the present report. None of the participants exhibited aberrant behavioral or physiologic responses during performance of these other protocols that could serve as grounds for exclusion from the present study. Importantly, the present results are generally consistent with previous non‐invasive studies in neurologically intact participants (Strauss et al. 2015; Shirazibeheshti et al. 2018).

There were multiple sources of variability that could have affected the results of the present study. Comparison between propofol (Nourski et al. 2018b) and the dexmedetomidine and sleep cohorts examined in the current study is confounded by differences in extent and type (electrocorticography vs. stereo‐EEG) of electrode coverage. In each of the two experiments presented in this study, there was variability across participants, both at baseline (awake state) and in the time course of dexmedetomidine sedation and the sleep experiment. The time course of dexmedetomidine sedation was affected by differences in drug administration, as deemed appropriate by the anesthesiologist. As a result, specific experimental conditions (awake, sedated in the dexmedetomidine experiment, awake, drowsy in the sleep experiment) are heterogeneous. Each experimental state encompassed a range of levels of alertness/arousal. Given the relatively small cohort sizes, sub‐dividing the data based on type of electrode coverage (electrocorticography vs. stereo‐EEG) was not practical.

Additionally, the three brain states examined in each of the two experiments are not pairwise equivalent. For instance, the awake state in the dexmedetomidine experiment likely represented a higher level of arousal than the awake state in the sleep experiment due to differences in the experimental environment (brightly lit operating room vs. dim monitoring room) and context (morning, time before the surgical procedure vs. afternoon, after lunch). To accomplish LOR, dexmedetomidine was administered in relatively high, albeit safe, doses of the drug, beyond what is commonly used for “arousable sedation.” As a result, the “unresponsive” state in the dexmedetomidine experiment was likely a lower level of arousal than “asleep.” Furthermore, recent intracranial work provides evidence that pharmacologically induced LOR and natural sleep are characterized by different neural dynamics, with changes more pronounced during anesthesia than sleep (Zelmann et al. 2023). Finally, unlike the dexmedetomidine experiment, where the presumed unconscious state was defined through a specific assessment criterion (OAA/S < 3), the presumed unconscious state “asleep” in the sleep experiment was more loosely defined.

The nature of the LGD task mandated analysis of data over the entire block duration (11 min in the present study; see Figure S1). Sedation and falling asleep are dynamic processes. Consequently, in each participant, each experimental block represented progressive state change rather than a steady state. Despite these multiple caveats, key findings previously reported for propofol (Nourski et al. 2018b) were replicated, including differences between changes in LD and GD responses. Likewise, similarities between the effects of dexmedetomidine and sleep on auditory predictive coding were observed despite the nuances of the respective experimental paradigms and associated variability of results. Finally, as experience grows through continued study of predictive coding during sleep, anesthesia, and pathological states of awareness, it can be expected that more accurate objective measures of these states will be developed and introduced into clinical settings.

### 4.6 Concluding Remarks

Overall, it can be argued that the LGD paradigm, despite its wide use to study conscious sensory processing, may be unnecessarily complicated given that GD effects are not a sensitive measure of LOC. Further, the paradigm is not practical either for the assessment of depth of anesthesia in the operating room environment or the assessment of disorders of consciousness in the intensive care unit. By contrast, simpler paradigms (e.g., roving oddball; Garrido et al. 2008; Boly et al. 2011) that probe short‐term novelty responses in a task‐independent and time‐efficient way may have great research and clinical utility for developing reliable biomarkers of LOC.

## Author Contributions

**Kirill V. Nourski:** conceptualization, data curation, formal analysis, funding acquisition, investigation, methodology, project administration, resources, software, supervision, validation, visualization, writing – original draft, writing – review and editing. **Mitchell Steinschneider:** methodology, writing – original draft, writing – review and editing. **Ariane E. Rhone:** investigation, project administration, writing – review and editing. **Rashmi N. Mueller:** investigation, methodology, resources, writing – review and editing. **Matthew I. Banks:** conceptualization, funding acquisition, investigation, methodology, project administration, writing – review and editing.

## Conflicts of Interest

The authors declare no conflicts of interest.

## Peer Review

The peer review history for this article is available at https://www.webofscience.com/api/gateway/wos/peer‐review/10.1111/ejn.70181.

## Supporting information


**Figure S1** LGD experimental paradigm. (a) Waveforms of the two vowel sounds /ɑ/ and /i/ used to construct the experimental stimuli. (b) Schematic of the four experimental stimuli. (c) Stimulus sequences. (d) Comparisons between trials to characterize local and global deviance effects. Modified from Strauss et al. (2015). Note that a modification of this paradigm was used in participants L525, L625, R720, R728 (dexmedetomidine experiment), and L372, L514, L585 (sleep experiment), wherein each sequence was preceded by a 15‐s instruction (“Press the button every time you hear this sound … Once again, press the button every time you hear this sound …”). In these participants, the number of GS and GD test trials in each sequence was reduced to 72 and 18, respectively, to maintain the same 11‐min duration of the recording block.
**Figure S2** Time course of dexmedetomidine (a,b) and sleep (c) experiments. (a) Time course of the dexmedetomidine experiment in each participant. Observer’s Assessment of Alertness/Sedation (OAA/S) scores (× symbols) and bispectral index (BIS) values (open circles) are plotted as functions of time. Dexmedetomidine infusion rates (in μg/kg/h) are shown underneath each plot. Bolus injections of dexmedetomidine (in μg/kg) are indicated by arrows. (b) Summary of OAA/S and BIS data from the 7 participants. OAA/S scores (left panel, crosses) and bispectral index (BIS) values (right panel, open circles) are plotted for each participant for three experimental blocks corresponding to the three studied arousal states. OAA/S scores represent average values of the two scores, obtained immediately before and after each block. BIS values are averages of minute‐by‐minute measurements within each 11‐min block; lower BIS values indicate more sedation. (c) Time course of the sleep experiment in each participant. In (a,c), vertical dashes denote button press behavioral responses to the target stimuli, rectangles represent data collection blocks.
**Figure S3** Example of changes in iEEG power spectra over the course of the sleep experiment in a representative participant (R413). (a) MRI side and ventral views of the hemispheric surface showing electrode coverage. Recording sites depicted as circles. PMC, premotor cortex; MFG, middle frontal gyrus; OG, orbital gyrus. (b) Top: time course of the sleep experiment, replotted from Figure S2, with the five blocks color‐coded. iEEG power in alpha (8–14 Hz) and beta (14–30 Hz) bands measured from three exemplar recording sites that did not exhibit significant responses to vowels or LGD effects. Blocks 1, 2, and 5 were used in data analysis as the awake, drowsy and asleep conditions, respectively.
**Figure S4** Changes in topography of AEP LD and GD effects during the dexmedetomidine experiment, shown for awake, sedated, and unresponsive state. Data from 7 participants, plotted in MNI coordinate space and projected onto FreeSurfer average template brain. Left hemisphere MNI *x*‐axis coordinates (*x*_MNI_) were multiplied by (−1) to map them onto the right‐hemisphere common space. Different symbol shapes denote participants. For each experimental block, the participant’s GD target hit rate in that block is denoted by the fill color. For each participant, change in symbol color from warmer to cooler across columns corresponds to a decline in task performance across the three blocks.
**Figure S5** Regional distribution and time course of LD and GD effects (panels a and b, respectively) during the dexmedetomidine experiment, shown for awake, sedated and unresponsive state (left, middle, and right column, respectively). Summary of data from 7 participants. Numbers of sites within each color‐coded ROI group exhibiting significant LD (a) and GD (b) effects are plotted as functions of time after the 5th vowel onset. 5th vowel onset for AEP and high gamma in upper and lower rows, respectively. The sole site with a high gamma GD effect in the unresponsive state was in the fusiform gyrus in participant R456. This site did not exhibit significant responses or deviance effects in the awake and sedated state, and thus this finding was interpreted as a false positive result.
**Figure S6** Changes in topography of AEP LD and GD effects during the sleep experiment, shown for awake, drowsy, and asleep state (columns 1–3). See caption of Figure S4 for details.
**Figure S7** Regional distribution and time course of responses to the first four vowels, LD and GD effects (panels a and b, respectively) during the sleep experiment, shown for awake, sedated and unresponsive state (left, middle, and right column, respectively). Summary of data from five participants. See caption of Figure S5 for details.
**Figure S8** Responses to local standard and deviant stimuli prior to and during induction of general anesthesia with dexmedetomidine in participant L525. (a) MRI reconstruction of the hemispheric surface and top‐down view of the superior temporal plane showing electrode coverage. Recording sites are depicted as circles, color‐coded by region‐of‐interest (ROI) group. Sites excluded from analysis due to excessive noise are denoted by “×.” Depth electrode insertion points are shown as black dots. (b) AEP waveforms (shades of cyan) and high gamma power envelopes (shades of magenta) recorded from six exemplar sites (callout boxes in panel a) in response to local standard and deviant stimuli. Lines and shading represent mean values and the 95% confidence intervals, respectively. Thick lines underneath response waveforms denote statistical significance (cluster‐based permutation tests, *p* < 0.05, FDR‐corrected). HGPM, Heschl’s gyrus, posteromedial portion; HGAL, Heschl’s gyrus, anterolateral portion; PT, planum temporale; STGP, superior temporal gyrus, posterior portion; STGM, superior temporal gyrus, middle portion.
**Figure S9** Responses to local standard and deviant stimuli during a sleep experiment in participant L372. (a) MRI reconstruction of the hemispheric surface and top‐down view of the superior temporal plane showing electrode coverage. Recording sites are depicted as circles, color‐coded by region‐of‐interest (ROI) group. Sites excluded from analysis due to excessive noise are denoted by “×.” Depth electrode insertion points are shown as black dots. (b) AEP waveforms (shades of cyan) and high gamma power envelopes (shades of magenta) recorded from six exemplar sites (callout boxes in panel a) in response to local standard and deviant stimuli. Lines and shading represent mean values and the 95% confidence intervals, respectively. Thick lines underneath response waveforms denote statistical significance (cluster‐based permutation tests, *p* < 0.05, false discovery rate‐corrected). HGPM, Heschl’s gyrus, posteromedial portion; STGP, superior temporal gyrus, posterior portion; STS, superior temporal sulcus; SMG, supramarginal gyrus.
**Table S1** Participant demographics and electrode coverage.

## Data Availability

The data used in this article will be made available by the authors upon request and after the establishment of a formal data sharing agreement.
