# Supplementary material for: Modulation of Auditory Novelty Processing by Dexmedetomidine and Natural Sleep: A Human Intracranial Electrophysiology Study
Source: Eur J Neurosci. 2025 Jul 13;62(1):e70181. doi: 10.1111/ejn.70181 (PMC12256162; doi:10.1111/ejn.70181)
Supplement: Supplementary file 1 — Figure S1 LGD experimental paradigm. (a) Waveforms of the two vowel sounds /ɑ/ and /i/ used to construct the experimental stimuli. (b) Schematic of the four experimental stimuli. (c) Stimulus sequences. (d) Comparisons between trials to characterize local and global deviance effects. Modified from Strauss et al. (2015). Note that a modification of this paradigm was used in participants L525, L625, R720, R728 (dexmedetomidine experiment), and L372, L514, L585 (sleep experiment), wherein each sequence was preceded by a 15‐s instruction (“Press the button every time you hear this sound … Once again, press the button every time you hear this sound …”). In these participants, the number of GS and GD test trials in each sequence was reduced to 72 and 18, respectively, to maintain the same 11‐min duration of the recording block. Figure S2 Time course of dexmedetomidine (a,b) and sleep (c) experiments. (a) Time course of the dexmedetomidine experiment in each participant. Observer’s Assessment of Alertness/Sedation (OAA/S) scores (× symbols) and bispectral index (BIS) values (open circles) are plotted as functions of time. Dexmedetomidine infusion rates (in μg/kg/h) are shown underneath each plot. Bolus injections of dexmedetomidine (in μg/kg) are indicated by arrows. (b) Summary of OAA/S and BIS data from the 7 participants. OAA/S scores (left panel, crosses) and bispectral index (BIS) values (right panel, open circles) are plotted for each participant for three experimental blocks corresponding to the three studied arousal states. OAA/S scores represent average values of the two scores, obtained immediately before and after each block. BIS values are averages of minute‐by‐minute measurements within each 11‐min block; lower BIS values indicate more sedation. (c) Time course of the sleep experiment in each participant. In (a,c), vertical dashes denote button press behavioral responses to the target stimuli, rectangles represent data collection blocks. Figure S3 Example of c [file EJN-62-0-s001.docx]

***Supplementary Information***

**Modulation of auditory novelty processing by dexmedetomidine and natural sleep:
A human intracranial electrophysiology study**

Kirill V. Nourski^1,2^, Mitchell Steinschneider^1,3^, Ariane E. Rhone^1^, Rashmi N. Mueller^1,4^,
Matthew I. Banks^5,6^

*^1^Department of Neurosurgery, The University of Iowa, Iowa City, IA 52242, USA
^2^Iowa Neuroscience Institute, The University of Iowa, Iowa City, IA 52242, USA
^3^Albert Einstein College Of Medicine, Bronx NY 10461, USA
^4^Department of Anesthesia, The University of Iowa, Iowa City, IA 52242, USA
^5^Department of Anesthesiology, University of Wisconsin, Madison, WI 53706, USA
^6^Department of Neuroscience, University of Wisconsin, Madison, WI 53706, USA*

**
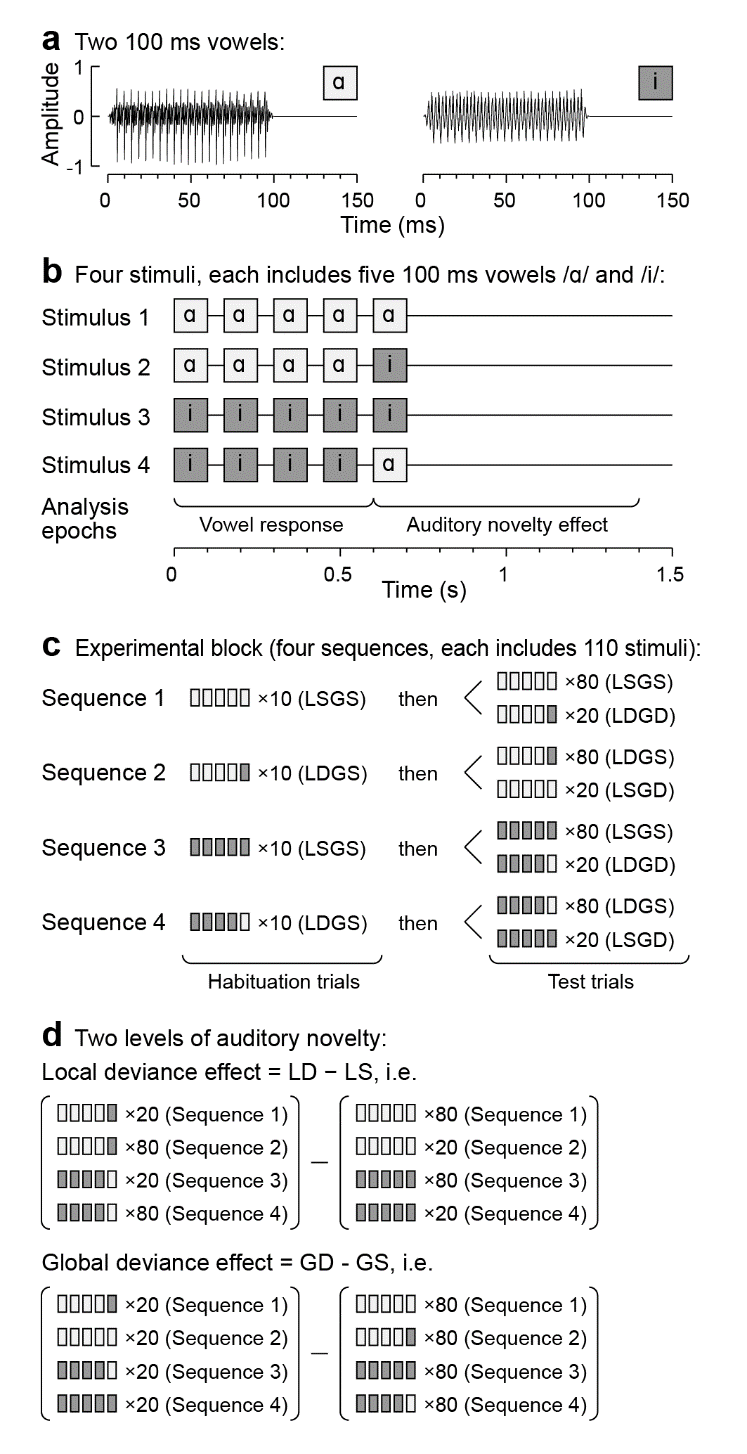
**

**Supplementary Figure 1:** LGD experimental paradigm. **a:** Waveforms of the two vowel sounds /ɑ/ and /i/ used to construct the experimental stimuli. **b**: Schematic of the four experimental stimuli. **c:** Stimulus sequences. **d:** Comparisons between trials to characterize local and global deviance effects. Modified from Strauss et al. (2015). Note that a modification of this paradigm was used in participants L525, L625, R720, R728 (dexmedetomidine experiment), and L372, L514, L585 (sleep experiment), wherein each sequence was preceded by a 15-second instruction (“*Press the button every time you hear this sound… Once again, press the button every time you hear this sound…*”). In these participants, the number of GS and GD test trials in each sequence was reduced to 72 and 18, respectively, to maintain the same 11-minute duration of the recording block.


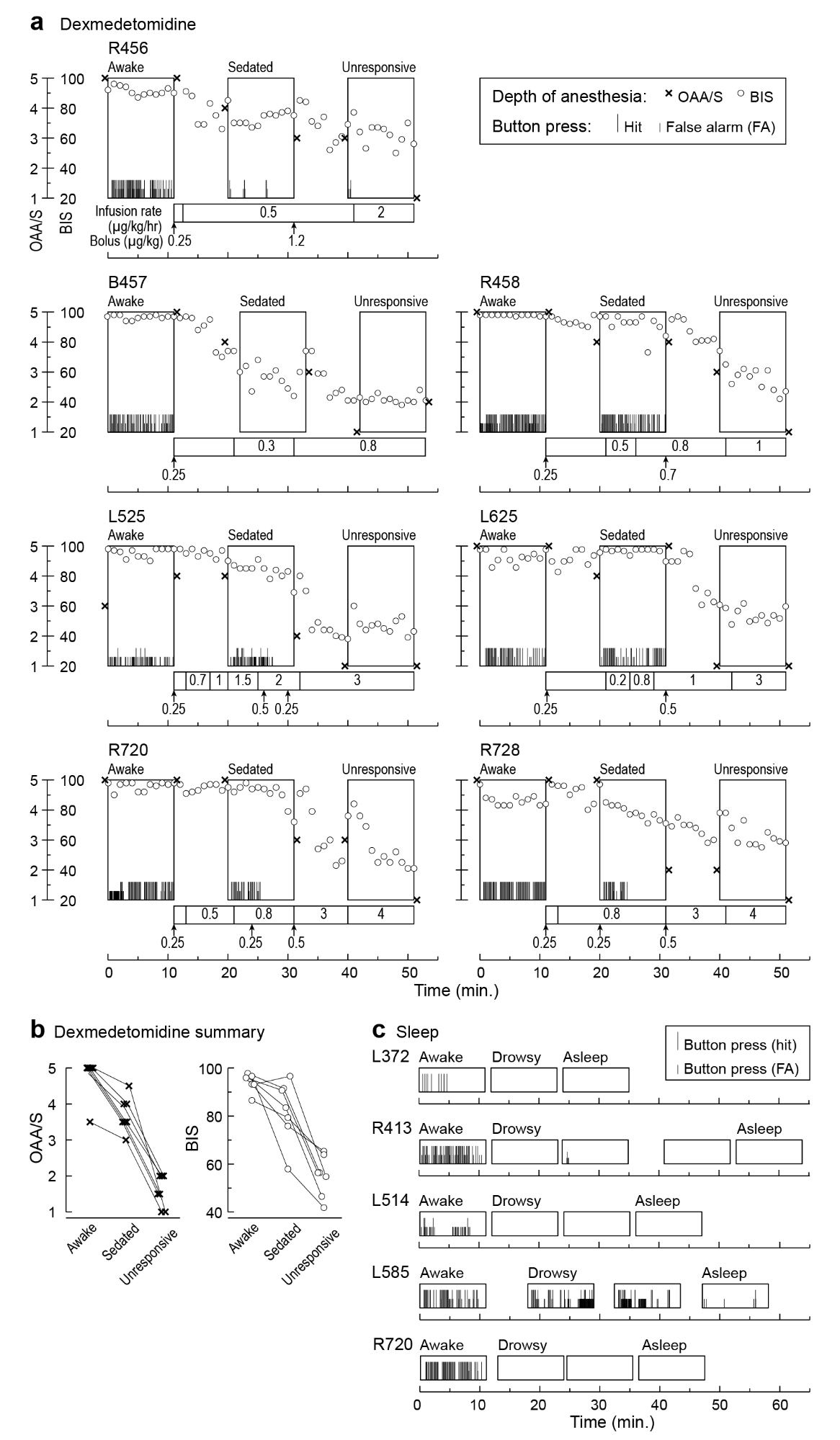


**Supplementary Figure 2.** Time course of dexmedetomidine **(a, b)** and sleep **(c)** experiments. **a:** Time course of the dexmedetomidine experiment in each participant. Observer’s Assessment of Alertness/Sedation (OAA/S) scores (× symbols) and bispectral index (BIS) values (open circles) are plotted as functions of time. Dexmedetomidine infusion rates (in µg/kg/hr) are shown underneath each plot. Bolus injections of dexmedetomidine (in µg/kg) are indicated by arrows. **b:** Summary of OAA/S and BIS data from the 7 participants. OAA/S scores (left panel, crosses) and bispectral index (BIS) values (right panel, open circles) are plotted for each participant for three experimental blocks corresponding to the three studied arousal states. OAA/S scores represent average values of the two scores, obtained immediately before and after each block. BIS values are averages of minute-by-minute measurements within each 11-min block; lower BIS values indicate more sedation. **c:** Time course of the sleep experiment in each participant. In panels **a** and **c**, vertical dashes denote button press behavioral responses to the target stimuli, rectangles represent data collection blocks.


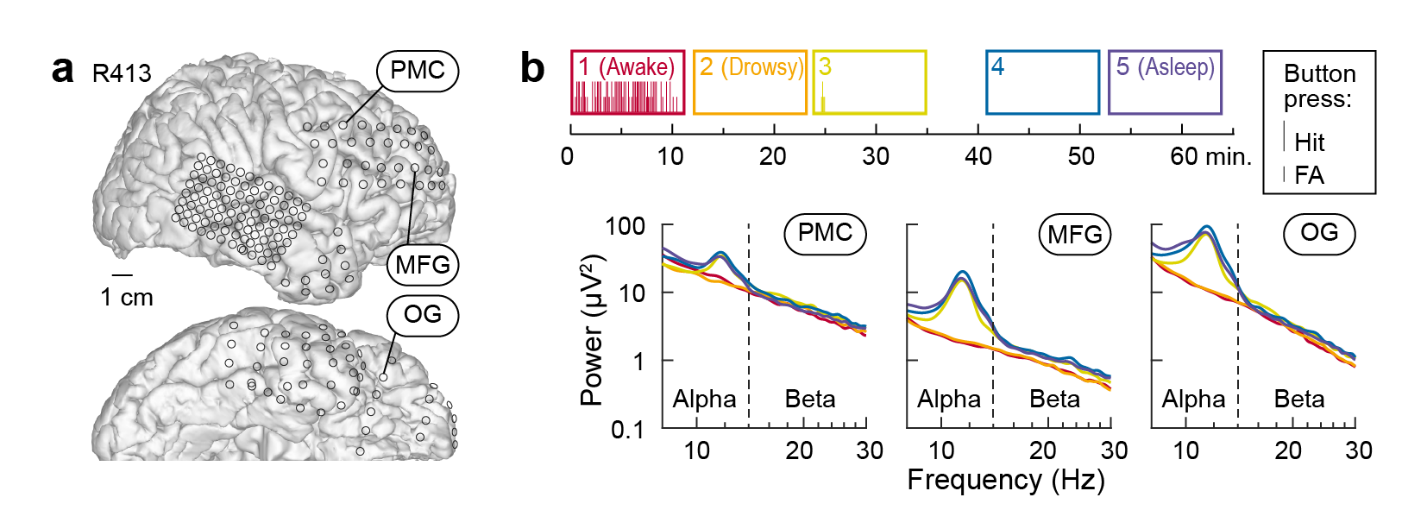


**Supplementary Figure 3.** Example of changes in iEEG power spectra over the course of the sleep experiment in a representative participant (R413). **a**: MRI side and ventral views of the hemispheric surface showing electrode coverage. Recording sites depicted as circles. PMC: premotor cortex; MFG: middle frontal gyrus; OG: orbital gyrus. **b:** Top: time course of the sleep experiment, replotted from Supplementary **Figure 2**, with the five blocks color-coded. iEEG power in alpha (8-14 Hz) and beta (14-30 Hz) bands measured from three exemplar recording sites that did not exhibit significant responses to vowels or LGD effects. Blocks 1, 2 and 5 were used in data analysis as the awake, drowsy and asleep conditions, respectively.


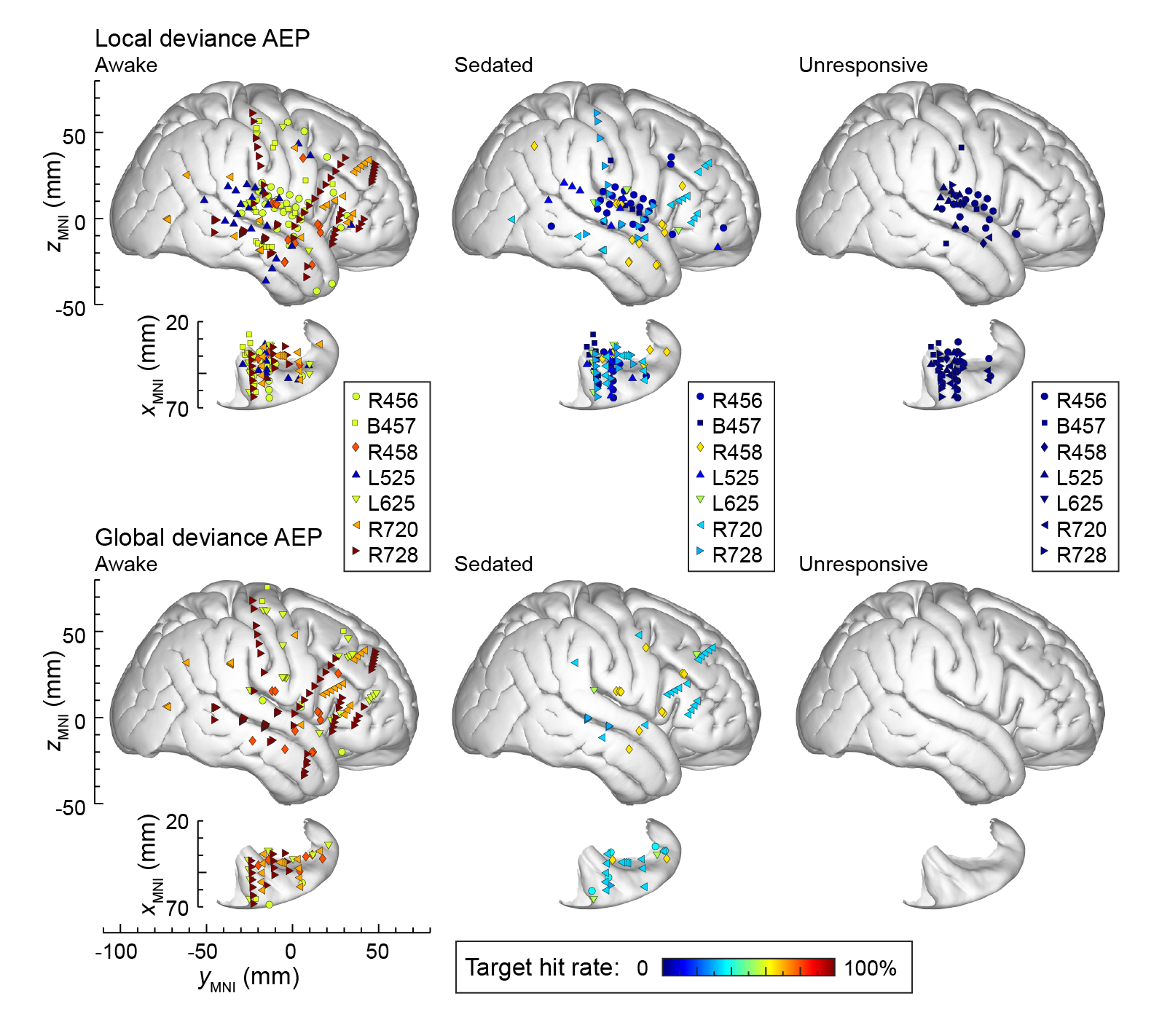


**Supplementary Figure 4.** Changes in topography of AEP LD and GD effects during the dexmedetomidine experiment, shown for awake, sedated and unresponsive state. Data from 7 participants, plotted in MNI coordinate space and projected onto FreeSurfer average template brain. Left hemisphere MNI *x*-axis coordinates (*x*_MNI_) were multiplied by (−1) to map them onto the right-hemisphere common space. Different symbol shapes denote participants. For each experimental block, the participant’s GD target hit rate in that block is denoted by the fill color. For each participant, change in symbol color from warmer to cooler across columns corresponds to a decline in task performance across the three blocks.


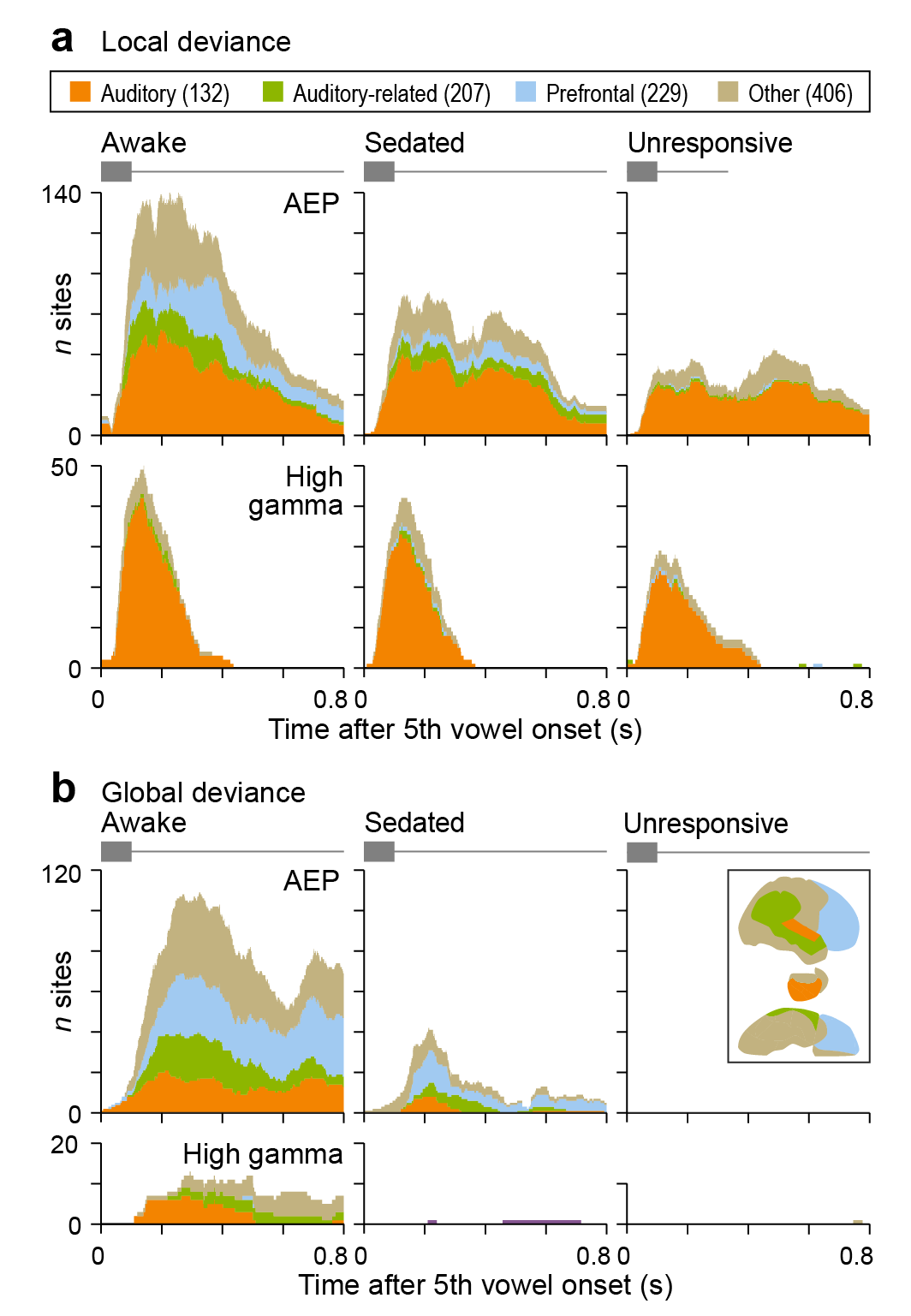


**Supplementary Figure 5.** Regional distribution and time course of LD and GD effects (panels **a**, and **b**, respectively) during the dexmedetomidine experiment, shown for awake, sedated and unresponsive state (left, middle and right column, respectively). Summary of data from 7 participants. Numbers of sites within each color-coded ROI group exhibiting significant LD (**a**) and GD (**b**) effects are plotted as functions of time after the 5th vowel onset. 5th vowel onset for AEP and high gamma in upper and lower rows, respectively. The sole site with a high gamma GD effect in the unresponsive state was in the fusiform gyrus in participant R456. This site did not exhibit significant responses or deviance effects in the awake and sedated state, and thus this finding was interpreted as a false positive result.

**
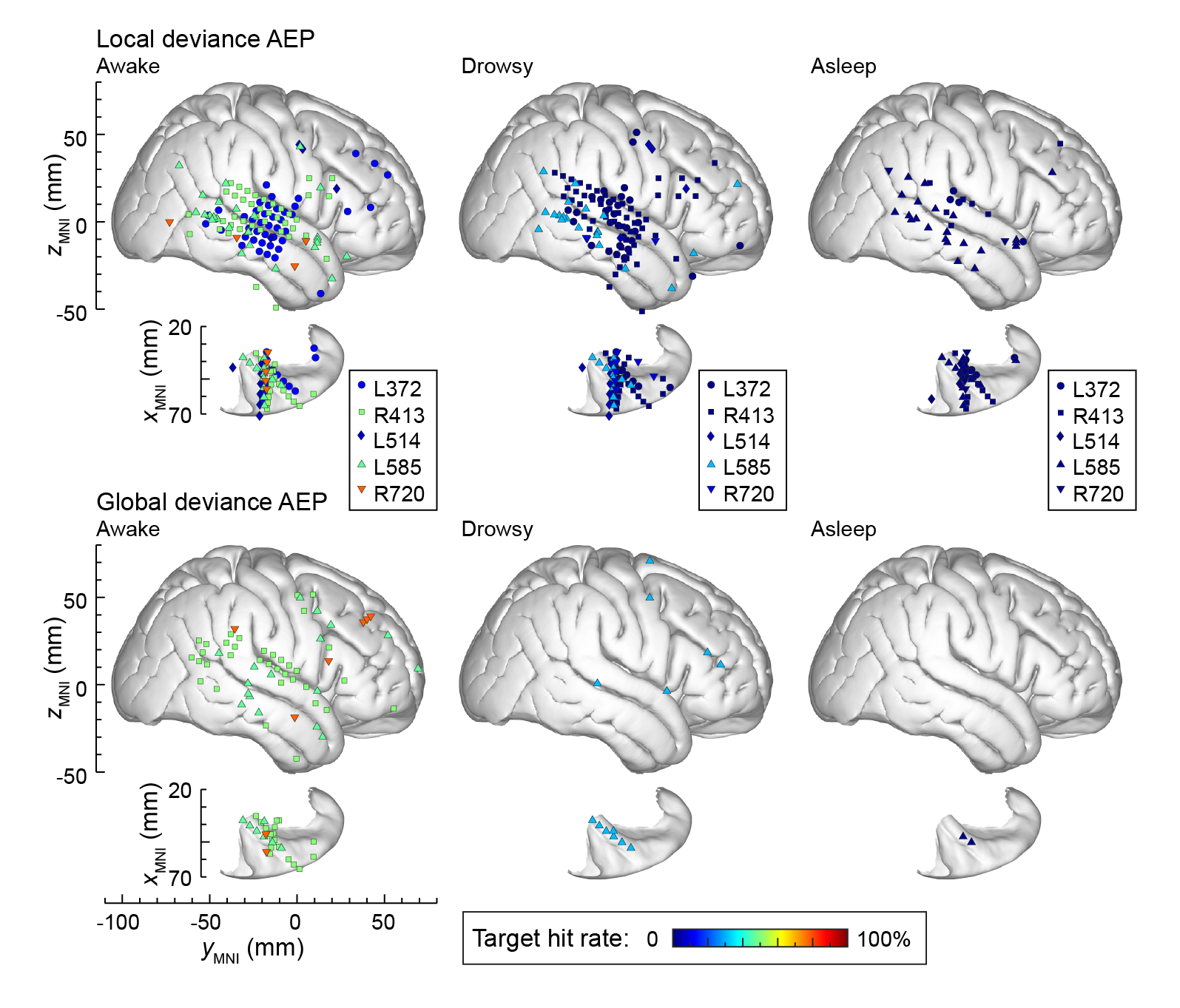
**

**Supplementary Figure 6.** Changes in topography of AEP LD and GD effects during the sleep experiment, shown for awake, drowsy and asleep state (columns 1-3). See caption of **Supplementary Figure 4** for details.


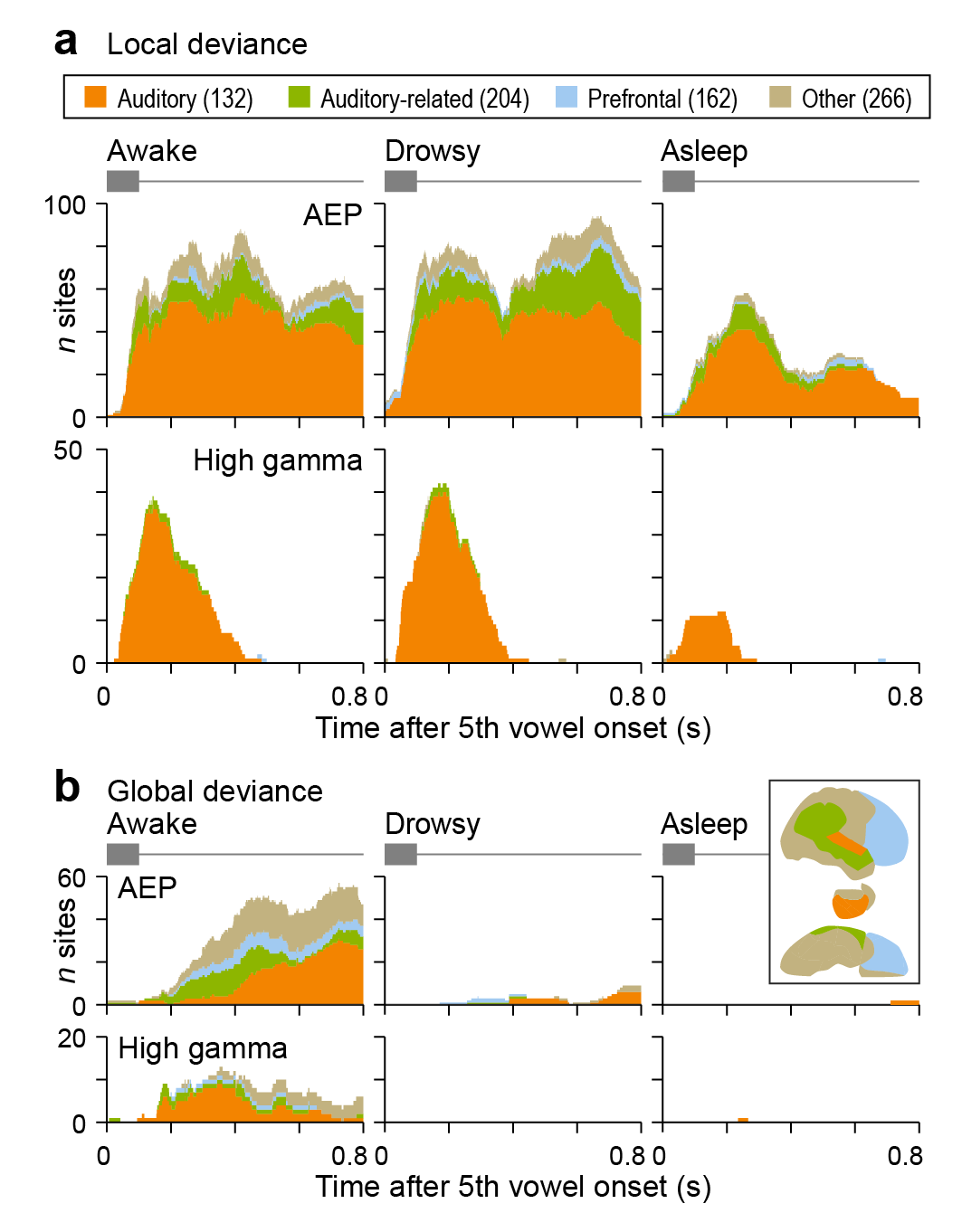


**Supplementary Figure 7.** Regional distribution and time course of responses to the first four vowels, LD and GD effects (panels **a** and **b**, respectively) during the sleep experiment, shown for awake, sedated and unresponsive state (left, middle and right column, respectively). Summary of data from 5 participants. See caption of **Supplementary** **Figure 5** for details.


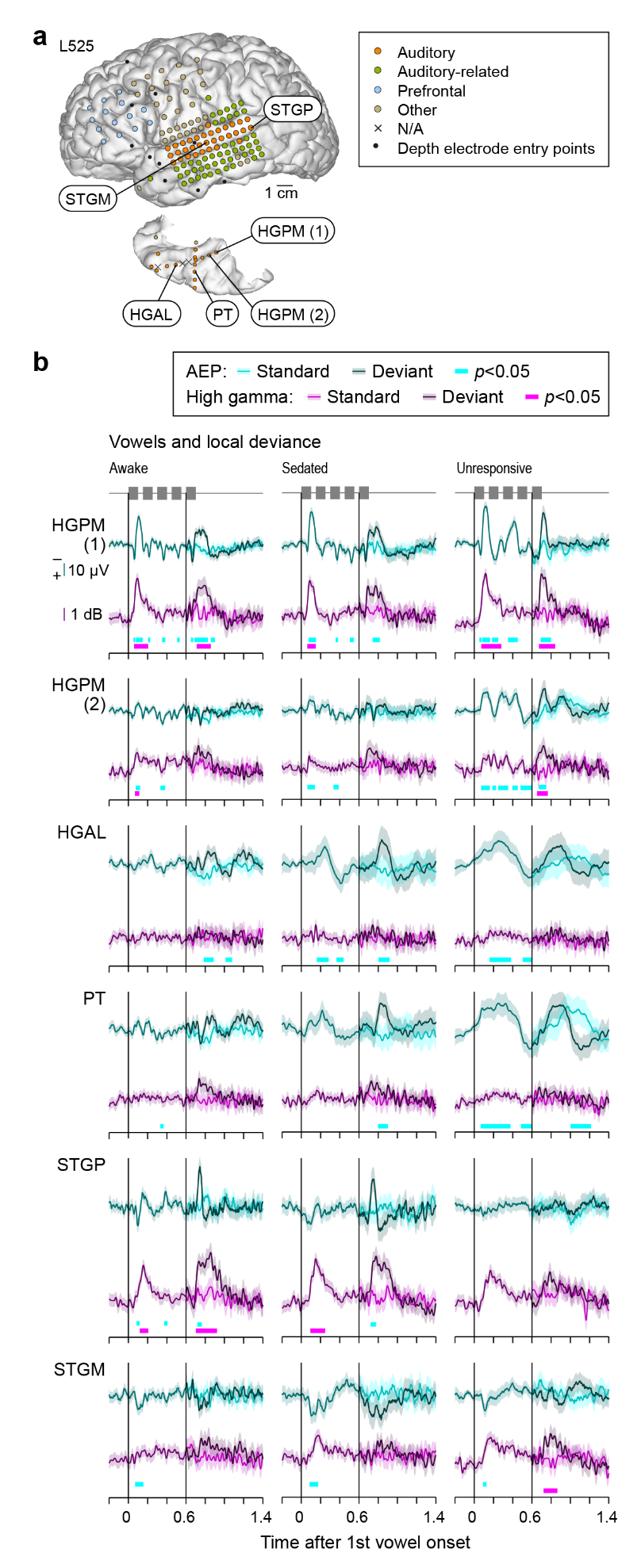


**Supplementary Figure 8.** Responses to local standard and deviant stimuli prior to and during induction of general anesthesia with dexmedetomidine in participant L525. **a**: MRI reconstruction of the hemispheric surface and top-down view of the superior temporal plane showing electrode coverage. Recording sites are depicted as circles, color-coded by region-of-interest (ROI) group. Sites excluded from analysis due to excessive noise are denoted by “×”. Depth electrode insertion points are shown as black dots. **b**: AEP waveforms (shades of cyan) and high gamma power envelopes (shades of magenta) recorded from six exemplar sites (callout boxes in panel **a**) in response to local standard and deviant stimuli. Lines and shading represent mean values and the 95% confidence intervals, respectively. Thick lines underneath response waveforms denote statistical significance (cluster-based permutation tests, *p*<0.05, FDR-corrected). HGPM: Heschl’s gyrus, posteromedial portion; HGAL: Heschl’s gyrus, anterolateral portion; PT: planum temporale; STGP: superior temporal gyrus, posterior portion; STGM: superior temporal gyrus, middle portion.


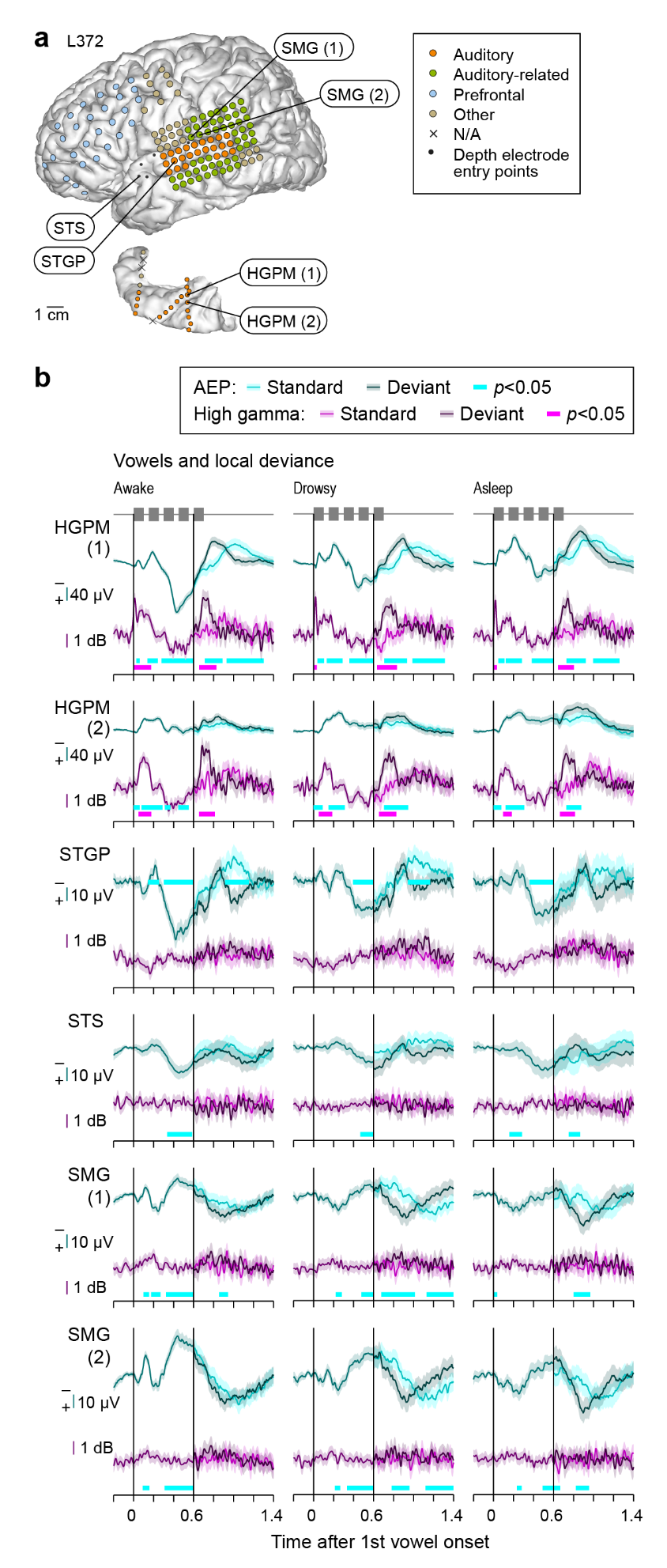


**Supplementary Figure 9.** Responses to local standard and deviant stimuli during a sleep experiment in participant L372. **a**: MRI reconstruction of the hemispheric surface and top-down view of the superior temporal plane showing electrode coverage. Recording sites are depicted as circles, color-coded by region-of-interest (ROI) group. Sites excluded from analysis due to excessive noise are denoted by “×”. Depth electrode insertion points are shown as black dots. **b**: AEP waveforms (shades of cyan) and high gamma power envelopes (shades of magenta) recorded from six exemplar sites (callout boxes in panel **a**) in response to local standard and deviant stimuli. Lines and shading represent mean values and the 95% confidence intervals, respectively. Thick lines underneath response waveforms denote statistical significance (cluster-based permutation tests, *p*<0.05, false discovery rate-corrected). HGPM: Heschl’s gyrus, posteromedial portion; STGP: superior temporal gyrus, posterior portion; STS: superior temporal sulcus; SMG, supramarginal gyrus.

**Supplementary Table 1.** Participant demographics and electrode coverage.

| Data set | Participant^a^ | Age (years) | Sex^b^ | Subdural arrays^c^ | Number of recording sites^d^ | | | | | Seizure focus |
| --- | --- | --- | --- | --- | --- | --- | --- | --- | --- | --- |
|  |  |  |  |  | Auditory | Auditory-related | Prefrontal | Other | Total |  |
| Dexmedetomidine | R456 | 31 | M | Y | 31 | 69 | 39 | 65 | 204 | R medial and lateral temporal |
|  | B457 | 18 | M | N | 20 | 12 | 27 | 52 | 111 | L medial temporal |
|  | R458 | 23 | M | N | 3 | 13 | 16 | 30 | 62 | R lateral temporal |
|  | L525 | 46 | F | Y | 47 | 55 | 34 | 83 | 219 | Bilateral multifocal |
|  | L625 | 24 | F | N | 9 | 4 | 20 | 55 | 88 | L medial temporal |
|  | R720^e^ | 42 | F | N | 8 | 22 | 19 | 24 | 73 | R medial temporal |
|  | R728 | 40 | M | N | 13 | 21 | 35 | 37 | 106 | R medial and lateral temporal |
| Sleep | L372 | 34 | M | Y | 44 | 58 | 35 | 54 | 191 | L temporal pole |
|  | R413 | 22 | M | Y | 45 | 78 | 45 | 55 | 223 | R medial temporal |
|  | L514 | 46 | M | N | 16 | 8 | 17 | 79 | 120 | L anterior insula |
|  | L585 | 39 | F | Y | 19 | 38 | 46 | 54 | 157 | L medial temporal |
|  | R720^e^ | 42 | F | N | 8 | 22 | 19 | 24 | 73 | R medial temporal |

^a^Letter prefix of the participant code denotes the side of electrode implantation over the presumed seizure focus (L = left; R = right; B = bilateral).

^b^F = female; M = male.

^c^Y = yes; N = no.

^d^The number of recording sites examined in the present study excluding recording sites identified as seizure foci, characterized by excessive noise and depth electrode contacts in white matter or outside the brain. See **Figure 1a** and **1b** for regional distribution of recording sites in the dexmedetomidine data set and sleep data set, respectively.

^e^R720 participated in both experiments and thus is included in both data sets.
